# MADNet: Marine Animal Detection Network using the YOLO platform

**DOI:** 10.1371/journal.pone.0322799

**Published:** 2025-05-08

**Authors:** Olarewaju Mubashiru Lawal, Yao Tan, Chuanli Liu

**Affiliations:** Sanjiang Institute of Artificial Intelligence & Robotics, Yibin University, Sichuan, China; MIT World Peace University Faculty for Engineering and Technology, INDIA

## Abstract

It is necessary to overcome the real-life challenges encountered in detecting marine animals in underwater bodies through computer vision for monitoring their populations and biological data. At the same time, the detectors for such tasks are limited by large parameters, high computation costs, low accuracy, low speed, and unfriendly deployment in low-power computing devices due to their large size. To tackle these problems, MADNet was developed using the YOLO framework, incorporating both anchor-based and anchor-free techniques. The structure of MADNet includes CBS, C3b, Bottleneck, SPPFr, and C3 modules, and it was evaluated against YOLOv5n, YOLOv6n, YOLOv7-tiny, and YOLOv8n with consistent application methods on various open-source underwater image datasets. Using the computation cost, trained time, loss, accuracy, speed, and mean absolute error (MAE) as performance evaluation metrics, the anchor-free methods performed better than the anchor-based methods. Similarly, the overall performance score for MADNet was analyzed at 27.8%, which is higher than 20% for YOLOv8n, 18.9% for YOLOv6n, 17.8% for YOLOv5n, and 15.6% for YOLOv7-tiny. As a result, MADNet is lightweight and effective for detecting marine animals in challenging underwater scenarios.

## Introduction

Marine animals, like fish, are aquatic species that rely on ocean habitats for survival. Meanwhile, regular sampling of marine animal populations is critical but challenging for monitoring trends in composition, size, relative abundance, and biomass in oceans and other bodies of water using underwater biological data. Marine biologists and conservationists need to be eager to use automated methods to address these issues rather than manual methods for sampling marine animals, which are time-consuming, damaging, and labor-intensive according to McLaren *et al*. [1]. Using computer vision and deep learning, a number of methods have been used to automatically detect and categorize marine animal in underwater videos. Salman *et al*. [2] used the LifeCLEF 2014 and LifeCLEF 2015 fish image datasets from the Fish4Knowledge repository to compare traditional machine learning methods with a deep learning technique. Mokhov [3], Choi [4], Jäger *et al*. [5], and Zhuang *et al*. [6] used LifeCLEF 2015 for fish detection and classification on underwater videos, which is based on the modular A* Recognition Framework (MARF), GoogleNet Convolution Neural Network, AlexNet deep CNN, and Single-Shot Multi-box Detector with ResNet-10, respectively. They were able to achieved good results. Nevertheless, low resolution, water murkiness, fish camouflage, dynamic backgrounds, aggregation of small targets, occlusions and jitters in imagery, different light conditions, similarity in shape and texture among different fish species, and other factors continue to impede the use of such automatic approaches in real-life scenarios, according to Jalal *et al*. [7] and Muksit *et al*. [8], which led to low detection accuracy. At the same time, the large parameters of the developed detection algorithms slow speed and pose a challenge for low-power computing device deployment [9].

The single-stage detector architecture known as the You Only Look Once (YOLO) has garnered significant attention over the years due to its exceptional performance in marine animal detection accuracy, particularly in regard to high speed and fewer parameters. The YOLO framework has moved from version to version due to continuous performance improvement. With the application of underwater vision, Sung *et al*. [10] enhanced the YOLO technique put forth by Redmon *et al*. [11] to report a speed of 16.7 frames per second (fps) and 93% classification accuracy for fish detection. Xu and Matzner [12] reported a mean average precision (mAP) of 53.92% using modified YOLO to monitor the effect of new technologies on fish and other wildlife in underwater video. YOLOv2, introduced by Redmon and Farhadi [13], is next to YOLO with the aim of improving detection accuracy significantly while making it faster. YOLOv2 was modified by Xia *et al*. [14] for a sea cucumber detection system. Redmon and Farhadi [15] later developed YOLOv3, which is more accurate than YOLOv2. Liu *et al*. [16] used a parallel correlation filter and YOLOv3 to create unique underwater fish detection and tracking algorithms, which were tested on the NVIDIA Jetson TX2 computing device in real-time. Jalal *et al*. [7] combined optical flow and Gaussian mixture algorithms with YOLOv3 to detect and classify fish in unconstrained underwater videos. They achieved F_1_-scores of 95.47% and 91.2%, as well as classification accuracies of 91.64% and 79.8% on the LifeCLEF2015 and University of Western Australia (UWA) datasets. YOLOv3 with MobileNetv1 proposed by Cai *et al*. [17] for fish detection in real-time breeding farm revealed average precision (AP) of 78.63% and speed of 13 fps. The modified YOLOv3 called YOLO-Fish proposed by Muksit *et al*. [8] recorded a low mAP of 76.56% for fish detection in unconstrained marine environments. To overcome the challenges of the underwater environment, the underwater detection algorithm introduced by Rosli *et al*. [18] based on YOLOv4 [19] achieved a remarkable 97.96% for mAP with speed of 46.6 fps. Hu *et al*. [20] used high-resolution feature maps with embedded dense units in YOLOv4 to achieve the detection of dense microparticles underwater, while NgoGia *et al*. [21] harnessed YOLOv4-tiny with Mosaic data augmentation to implement a real-time cultured sea cucumber detector on the autonomous underwater vehicle (AUV). Shi *et al*. [22] stated that the Marine Organism Detection Algorithm (MODA), which is based on modified YOLOv4-tiny, improved mAP from 74% to 76.62% and 92.37% to 98.41% on the Underwater Robot Picking Contest (URPC) for 2020 and Aquarium datasets, respectively. Zhang *et al*. [23] developed an improved YOLOv4 using a new convolution module and network structure in response to the complex underwater environment, achieving 91.1% of mAP with a detection speed of 58.1 fps. Following YOLOv4, Jocher *et al*. [24] proposed YOLOv5, which aims to achieve fewer parameters with high speed. For this reason, Yoshida *et al*. [25] suggested a simple YOLOv5-based monitoring system for small sea cucumbers grown in cages on the seafloor. Li *et al*. [26] then replaced YOLOv5’s backbone with a lightweight network using group convolution and inverse residual block for recognizing underwater scallops. Wang *et al*. [27] applied the YOLOv5 baseline for underwater object detection and reported it to be lightweight, fast, accurate, and suitable for the underwater environment. To perform effective underwater target detection, Liang *et al*. [28] introduced a lightweight detection algorithm based on YOLOv5, which employs depth-wise separable convolution and achieves a mAP of 85.1%. The algorithm resulted in a 39% reduction in parameters and operated at a speed of 85 fps on the URPC2021 dataset. Liu *et al*. [29] added a convolutional block attention module (CBAM) to the backbone of YOLOv5 for feature extraction, which was evaluated on the URPC2021 dataset, reaching a mAP of 79.2%. Similarly, the proposed MAD-YOLO [30] for marine benthos detection that is validated on the URPC2020 dataset increases the mAP of YOLOv5 from 49.8% to 53.4%. Wang *et al*. [31] reported a lightweight underwater target detection based on the YOLOX [32], which was evaluated on the URPC2020 dataset, and its detection accuracy increased to 82.69%. In the case of YOLOv6 [33], a mAP of 83.5% and speed of 64 fps were revealed on the URPC2021 dataset, and mAP of 95.8% and speed of 86 fps were reported on the brackish dataset [34]. Wang *et al*. [35] modified YOLOv6 to develop YOLOv6-ESG, focusing on the detection of seafood underwater with the URPC2022 dataset. By integrating EfficientNetv2 [36], ODConv [37], and SPD-Conv [38] into the backbone, along with GSConv and VoVGSCSP [39] into the neck section of YOLOv6-ESG, they achieved a mean average precision (mAP) of 86.6%, a reduction of 75.44% in parameters, and a processing speed of 50.66 fps. Wang *et al*. [40] have shown that the detection abilities of YOLOv7 are better than those of YOLOv4 and YOLOv5. According to Shankar and Muthulakshmi [41], YOLOv7 also had greater accuracy than YOLOv3 and YOLOv5 when detecting marine species in water, though it operated at a slower speed. Liu *et al*. [34] created YOLOv7-AC to detect targets underwater, which performs better than standard YOLOv7 by utilizing ResNet-ACmix, ACmixBlock, and the Global Attention Mechanism. Tested on the URPC2021 as well as brackish datasets, YOLOv7-AC achieved mAP scores of 89.6% and 97.4%, with processing speeds of 74 and 92 fps, respectively. Yu *et al*. [42] introduced a new network called U-YOLOv7, which is based on YOLOv7 and aims to detect underwater life. A gain of 3.2% in accuracy, 2.3% in recall, and 2.8% in mAP, achieving 179 fps, was found when compared to YOLOv7. Liu *et al*. [43] expanded on YOLOv7 by incorporating CBAM and achieved mAP of 94.4% for detecting rockfish, marking an increase of 3.5% over YOLOv7. However, the YOLO versions mentioned earlier rely on anchor-based detectors. Jocher *et al*. [44] launched YOLOv8 to improve object detection performance by adopting anchor-free methods. YOLOv8 was created with new capabilities that surpass earlier versions. Zhang *et al*. [45] assessed the URPC2019 and URPC2020 datasets and recorded mAP results of 76% and 78.3% for YOLOv8, while the enhanced YOLOv5 had results below 79.8% and 79.4%. Currently, investigations into marine animal detection focusing on YOLOv8 are limited, as is the application of anchor-free techniques in other mainstream YOLO variants for comparative performance analysis. Moreover, improving the performance of marine animal detection is imperative.

To reach goals of high accuracy, high speed, fewer parameters, low computing expenses, and lightweight for friendly algorithm deployment on low-power devices, and to ensure stability in challenging underwater conditions, the anchor-based YOLOv5 and anchor-free YOLOv8 have been chosen for enhancement. This has led to the development of MADNet, designed to address difficulties encountered in real-world scenarios. The success of the suggested algorithm has been proven through tests performed on available underwater image datasets, specifically Brackish, UOv2, and RUOD. The contributions to knowledge of this paper are to:

(1) introduce modules of three activated convolutional layers with a Bottleneck (C3b) and Spatial Pyramid Pooling Faster (SPPFr) into the backbone network and replace the C2f of the YOLOv8 head network with C3 to develop MADNet for marine animal detection.
(2) create MADNet that is reduce in computational cost, fewer in parameters, accurate, real-time fast, robust, and deployable-friendly.
(3) compare the performance score of MADNet to that of YOLOv5n, YOLOv6n, YOLOv7-tiny, and YOLOv8n algorithms using anchor-based and anchor-free methods.

The rest of this paper, in the second section, focuses on the YOLO platform in relation to YOLOv5 for anchor-based and YOLOv8 for anchor-free, while the third section explains the underwater datasets, proposed MADNet, and evaluations. The fourth section is for results and discussion, and the fifth section summarizes the conclusions.

## YOLO platform

### YOLOv5

**Fig 1:**
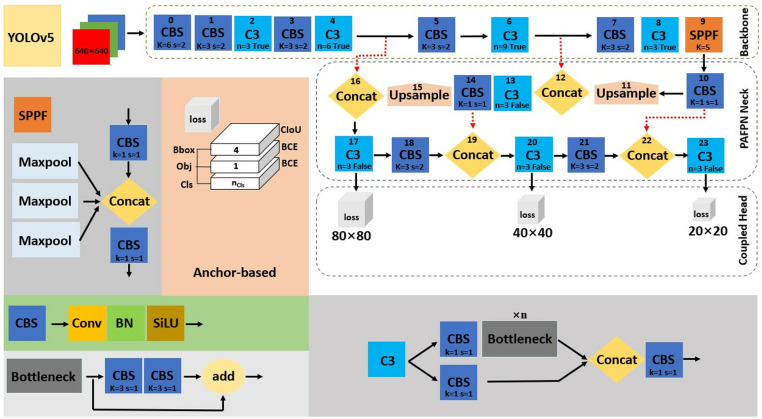
The anchor-based network of YOLOv5 [24].


The anchor-based architecture of YOLOv5 [24] displayed in Fig 1 is divided into the input, backbone, neck, and head networks. The input consists of adaptive anchor, mosaic data augmentation, and adaptive image scaling (e.g., n: 0.33 depth and 0.25 width). The backbone, comprising convolution-batch normalization-SiLU (CBS), three CBS (C3), and Spatial Pyramid Pooling Fast (SPPF) modules, is utilized for the aggregation of fine-grained images and feature map extraction. The neck for multiscale feature fusion applied the Path Aggregation with Feature Pyramid Network (PAFPN). It combines the extracted feature maps collected by the backbone network before passing the integrated feature maps to the head network. The head network, which derives predictions from the anchor boxes for object detection, employs a coupled head and outputs the category probability of the object target, score and location of the bounding box. The coupled head uses a complete intersection-over-union (CIoU) loss function [46] for bounding box (Bbox) and binary cross-entropy (BCE) loss for classification (Cls) and objectness (Obj). The CIoU loss mentioned in Eq. (1) improves convergence speed and localization accuracy, where S represents the overlap area, D denotes the centroid distance, V indicates the aspect ratio, B is the predicted box, and B^gt^ is the real box. According to Lawal *et al*. [47], BCE loss is stated by Eq. (2) as y being the label for the output range (0–1) through sigmoid, and p(y) is the predicted probability for all N points.

$$L_{\text{CIoU}}\text{ = S}\left(\text{B, }{\text{B}}^{\text{gt}}\right)+\text{D}\left(\text{B, }{\text{B}}^{\text{gt}}\right)+\text{V}\left(\text{B, }{\text{B}}^{\text{gt}}\right)$$ (1)

$$\text{BCE = }-\frac{\text{1}}{\text{N}}\sum_{\text{i-0}}^{\text{N}}{\text{y}}_{\text{i}}\text{*log}\text{(p(}{\text{y}}_{\text{i}}\text{)) + (1-}{\text{y}}_{\text{i}}\text{)*log(1-p(}{\text{y}}_{\text{i}}\text{)) }$$ (2)

### YOLOv8

**Fig 2:**
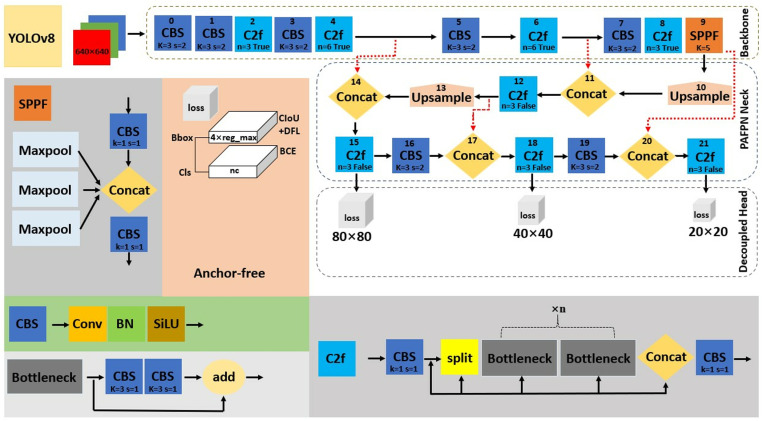
The anchor-free network of YOLOv8 [44].


Just like the YOLOv5 operation, the anchor-free architecture of YOLOv8 [44] shown in Fig 2 is divided into the input, backbone, neck, and head networks. The input consists of mosaic data augmentation and adaptive image scaling (e.g., n: 0.33 depth and 0.25 width) without adaptive anchor. The backbone adds CBS, two CBS with two expandable Bottlenecks (C2f), and SPPF modules for the extraction of feature maps, as depicted in Fig 2. The neck network, which includes CBS and C2f modules, used PAFPN for multiscale feature fusion. The head network is decoupled, having a CIoU loss function (see Eq. (1)) with distribution focal loss (DFL) [48] and BCE loss (see eq. (2)) for Bbox and Cls, respectively. Meanwhile, DFL is defined by Eq. (3) as s_i_ is the output of sigmoid, y is the label, and y_i_ and y_i+1_ represent the intervals. It enables probability density and distribution close to the target location.

$$\text{DF}{\text{L}}_{\text{(}{\text{s}}_{\text{i}}\text{,}{\text{s}}_{\text{i+1}}\text{)}}\text{ = -((}{\text{y}}_{\text{i+1}}\text{-y)log(}{\text{s}}_{\text{i}}\text{) + (y-}{\text{y}}_{\text{i}}\text{)log(}{\text{s}}_{\text{i+1}}\text{)) }$$ (3)

## Materials and methods

### Underwater datasets

#### Brackish.

**Fig 3:**
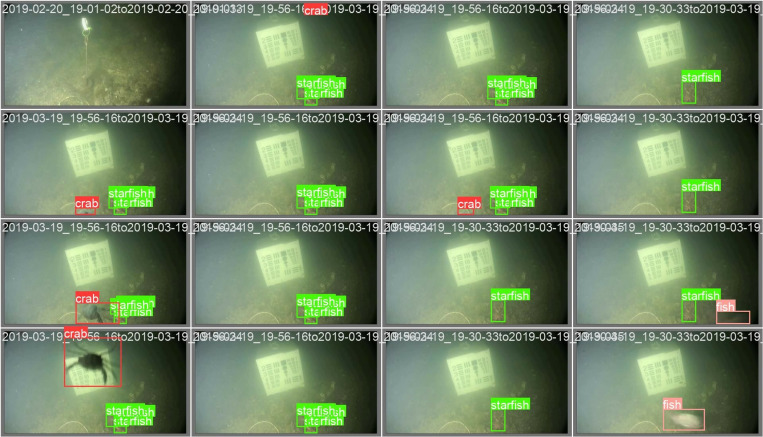
The samples of annotated images in the brackish dataset [49].

The brackish is a publicly available dataset created by Pedersen *et al.* [49] from Aalborg University. A camera mounted 9 meters below the surface on the Limfjord Bridge in northern Denmark was utilized to obtain the image dataset. The brackish processed dataset with annotations provided by Roboflow that is readily available for public utilization was harnessed in the paper. The dataset with 14674 images, including crab, fish, jellyfish, shrimp, small fish, and starfish, was randomly divided into train-set, valid-set, and test-set in an 8:1:1 ratio, with 11739, 1467, and 1468 images, respectively. Some of the sample images annotated in the dataset are shown in Fig 3. For having the bounding box annotations of 28518, 3581, and 3466 from the train-set, valid set, and test-set, respectively, the number of targets is most abundant in the crab with 12348, followed by small fish with 10768, starfish with 7912, fish with 3352, jellyfish with 637, and shrimp with 548.

#### UOv2.

**Fig 4:**
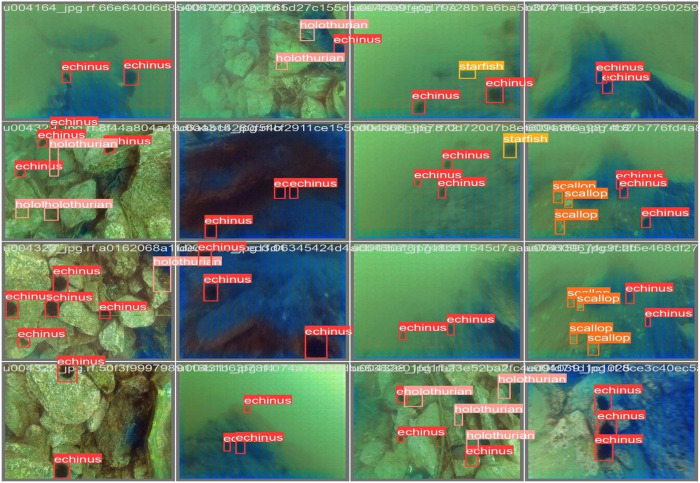
The samples of annotated images in the UOv2 dataset [50].

The studied underwater objects version two (UOv2) dataset was publicly provided by Roboflow100 [50]. However, this dataset, which was originally collected by the 2019 China Underwater Robot Professional Contest (URPC2019), consists of 7600 optical images captured in real marine environments with various scales and lighting conditions. Fig 4 indicates some of the annotated images in the dataset. Five target categories from various underwater scenarios are included in the dataset, namely echinus, holothurian, scallop, starfish and waterweeds. Roboflow100 cleaned the dataset to ensure data quality and accuracy, and randomly divided it into 5320 train-set, 1520 valid-set, and 760 test-set in a 7:2:1 ratio for subsequent experiments, where bounding box annotations of 37158, 10480, and 5046 were extracted, respectively.

#### RUOD.

**Fig 5:**
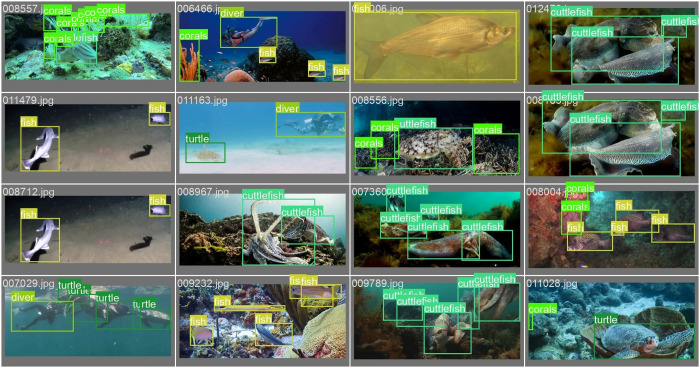
The samples of annotated images in the RUOD dataset [51].

The Rethinking General Underwater Object Detection (RUOD) image dataset created by Fu *et al*. [51] was also experimented with to evaluate the general underwater scene. With reference to various complex marine objects and diverse environmental challenges such as haze-like effect, color cast, and light interference, RUOD contains 14000 images with 74903 bounding boxes and 10 target categories, namely holothurian, echinus, scallop, starfish, fish, corals, diver, cuttlefish, turtle, and jellyfish, having image proportions of 10.2%, 15.1%, 9.9%, 10.8%, 17.5%, 11.9%, 8.5%, 7.1%, 5.5%, and 3.5%, respectively. Some of the annotated images contained in the dataset are displayed in Fig 5. Meanwhile, the dataset is randomly divided into 9800 images of 51934 annotations and 4200 images of 22969 annotations for the train-set and valid-set, respectively. Similarly, the 100 images each for blur, color, and light, totaling 300 images provided, are taken as a test-set in this paper.

#### MADNet.

**Fig 6:**
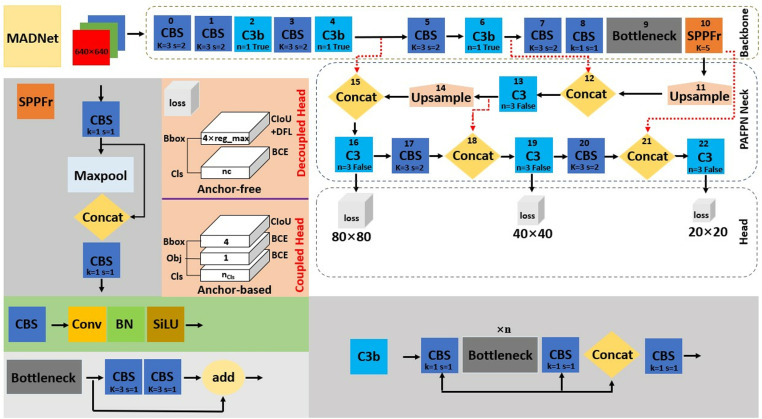
The network of MADNet: For anchor-based and anchor-free.

The MADNet designed as shown in Fig 6 for marine animal detection builds upon the YOLO platform with inspiration drawn from improved YOLOv5s by Lawal *et al*. [52]. It is produced as anchor-based and anchor-free using YOLOv5 and YOLOv8, respectively, to enable detection performance comparisons. MADNet contains the input, backbone, neck, and head networks as displayed in Fig 6. The input aspect of an anchor-based network integrates adaptive anchor, mosaic data augmentation and adaptive image scaling of 0.33 depth and 0.25 width, while the input aspect of an anchor-free network, which has others, is without adaptive anchor. The backbone network, which is a convolutional neural network to accumulate fine-grained images and extract feature maps, is similar in both anchor-based and anchor-free networks. As shown in Fig 6, the backbone includes CBS, three activated convolutional layers with a Bottleneck (C3b), Bottleneck and Spatial Pyramid Pooling Faster (SPPFr) modules. The CBS is a convolution layer activated with SiLU that is used for downsampling feature maps. The introduced C3b module for feature map extraction embedded the feature concatenation of two CBS with Bottleneck in between them, followed by another CBS at the end, as depicted in Fig 6. Feature concatenation facilitates the exchange of information between complementary features of the upper and lower layers, as stated by Lawal *et al*. [52]. The purpose of the C3b module was to lower the computation costs and number of parameters while maintaining detection accuracy and improving speed. The Bottleneck, which consists of two CBS, enables more learning of features towards increased accuracy. In place of the SPPF module adopted by YOLOv5 and YOLOv8, SPPFr was designed and incorporated into the backbone network to speed up the computation and reduce the loss of features during learning while maintaining enhancement of feature expression ability. As indicated in Fig 6, SPPFr was designed to have a single maxpooling concatenated with a CBS. For the neck network with the different scale targets, both anchor-based and anchor-free networks used the same PAFPN module. As shown in Fig 6, the PAFPN arrangement of MADNet is similar to that of YOLOv8 shown in Fig 2, except for the C2f module that is replaced by the C3 module of YOLOv5 in Fig 1. The C3 module was selected instead of the C2f module so as to lower the number of parameters. The C3 enables excellent feature extraction while curbing gradient information duplication. Meanwhile, anchor-based and anchor-free are associated with coupled and decoupled head networks, respectively. The coupled head network is from YOLOv5, and the decoupled head is from YOLOv8, and their losses are defined by Eq. (1) – (3). At different scales from the neck, the head allows MADNet to detect large, medium and small targets within an image.

#### Experiment and evaluation.

With reference to anchor-based and anchor-free methods, the experiments of MADNet, including the compared YOLOv5n, YOLOv6n, YOLOv7-tiny and YOLOv8n, were conducted on underwater datasets using the YOLO platform, as detailed in Table 1. The algorithms were trained from scratch using random initialization with a 640 × 640 × 3 input image, 32 batches, 300 epochs, and other default settings.

**Table 1:** The hardware and environment details.

Hardware	Configure	Environment	Version
System	Ubuntu20.04	Python	3.8.16
CPU	Core i7-12700F	Conda	23.1.0
GPU	RTX5000 (16G)	PyTorch	1.12.1
RAM	32G	CUDA	11.3.1
Hard-disk	2.0T	CUDNN	8.8.0

The trained algorithms were evaluated on valid-set and test-set using the common metrics stated in Eq. (4–10), respectively, for precision (P), recall (R), average precision (AP), mean average precision (mAP), number of parameters (params), floating-point operations per second (FLOPs) and speed (fps). The true positive (TP) is for correct detections, the false negative (FN) is for missed detections, and the false positive (FP) is for incorrect detections, P_(R)_ represents the maximum P value when the R is greater than or equal to R (R ranges from 0 to 1), AP is the area under curve based on single-class, mAP is the mean AP values over multi-class, C is the total number of classes, i is the input-size, j is the serial number, k is the convolution kernel-size, o is the output size, and H × W (Height and Width) is the size of the outputted feature map. Layer is the network topology, params (10^6^) is the number of trainable parameters, and FLOPs measure the number of floating-point arithmetic operations. The speed measures the real-time in frames per second (fps). T_pre_ is the preprocessing time, T_infer_ is the inference time, and T_post_ is the post-processing time, which are computed in milliseconds (ms).


$$\text{P = }\frac{\text{TP}}{\text{TP+FP}}$$ (4)

$$\text{R = }\frac{\text{TP}}{\text{TP+FN}}$$ (5)

$$\text{AP=}\int_{\text{0}}^{\text{1}}{\text{P}}_{\text{(R)}}\text{dR}$$ (6)

$$\text{mAP = }\frac{\sum {\mathrm{\backslash nolimits}}_{\text{j=1}}^{\text{C}}\text{A}{\text{P}}_{\text{j}}}{\text{C}}$$ (7)

params = [i×(k×k)×o]+o (8)

FLOPs = H×W×params  (9)

$$\text{speed (fps) = }\frac{\text{1000}}{{\text{T}}_{\text{pre}}\text{+}{\text{T}}_{\text{infer}}\text{+}{\text{T}}_{\text{post}}}$$ (10)

The mean absolute error (MAE) stated in Eq. (11) quantifies the accuracy of the algorithm toward better counting performance with lower values. The n is the number of images, while G_i_ and P_i_, respectively, represent the ground-truth count and predicted count for the i-th image.


$$\text{MAE =}\frac{\text{1}}{\text{n}}\sum {\mathrm{\backslash nolimits}}_{\text{i=1}}^{\text{n}}\left|{\text{G}}_{\text{i}}\text{-}{\text{P}}_{\text{i}}\right|$$ (11)

## Results and discussions

### Computation cost

The computational cost presented in Table 2 and Table 3 are necessary to measure the amount of resources the developed algorithms use in training. The params, size, and FLOPs exhibited a consistent trend, thereby substantiating Eq. (9), except for the layers. The increased layers in the anchor-free algorithms are a trade-off for their flexibility, while the fewer layers in the anchor-based algorithms leverage predefined anchors to simplify. Using the least number of layers, the simplicity of the network was noted in YOLOv6n, followed by MADNet and other algorithms. MADNet is computationally inexpensive compared to YOLOv8n [44], YOLOv5n [24], YOLOv6n [33], and YOLOv7-tiny [40] for having the smallest params, size, and FLOPs. For this reason, the execution time required for training the brackish, UOv2 and RUOD datasets is smaller, as shown in Table 2 and Table 3. At the same time, the trained time for the dataset of UOv2 is less than brackish and RUOD due to the number of images. Therefore, MADNet for marine animal detection is efficient enough to run in a reasonable amount of time. Meanwhile, the computational costs of anchor-free algorithms in Table 3 are higher than those of anchor-based algorithms in Table 2, and interestingly, the trained time of anchor-free algorithms is lower than that of anchor-based algorithms. This is to say that the anchor-based methods are candid for computation cost, while the anchor-free methods are best for training time.

**Table 2:** Computational cost of anchor-based algorithms.

Algorithms (Anchor-based)	Layers	Params (×10^6^)	Size (×10^6^)	FLOPs (G)	Trained time (hours)		
**Brackish**	**UOv2**	**RUOD**
YOLOv5n	214	2.2	4.8	5.3	12.845	3.898	14.383
YOLOv6n	147	3.9	8.1	10.3	12.172	3.377	13.616
YOLOv7-tiny	201	6.0	12.4	13.1	12.687	4.089	14.378
YOLOv8n	177	2.3	5.2	5.2	12.500	3.385	13.672
MADNet	176	1.8	3.9	5.1	11.850	3.259	13.269

**Table 3:** Computational cost of anchor-free algorithms.

Algorithms (Anchor-free)	Layers	Params (×10^6^)	Size (×10^6^)	FLOPs (G)	Trained time (hours)		
					Brackish	UOv2	RUOD
YOLOv5n	262	2.5	5.3	7.1	7.897	4.175	8.916
YOLOv6n	195	4.2	8.7	11.8	7.038	3.578	7.713
YOLOv7-tiny	249	8.1	16.5	21.2	9.132	4.603	9.986
YOLOv8n	225	3.0	6.2	8.1	7.080	3.604	8.015
MADNet	224	2.3	4.8	7.6	6.608	3.410	7.596

### Training loss

**Fig 7:**
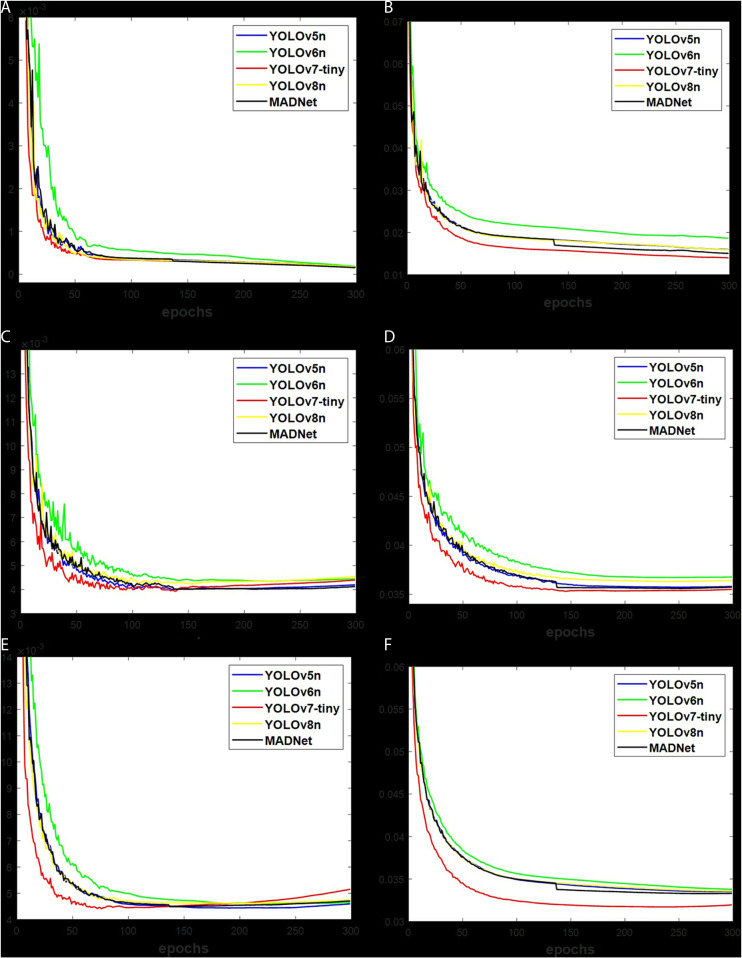
The anchor-based validation loss for (a) Brackish-Clsloss, (b) Brackish-Bbloss, (c) UOv2-Clsloss, (d) UOv2-Bbloss, (e) RUOD-Clsloss, and (f) RUOD-Bbloss.

**Fig 8:**
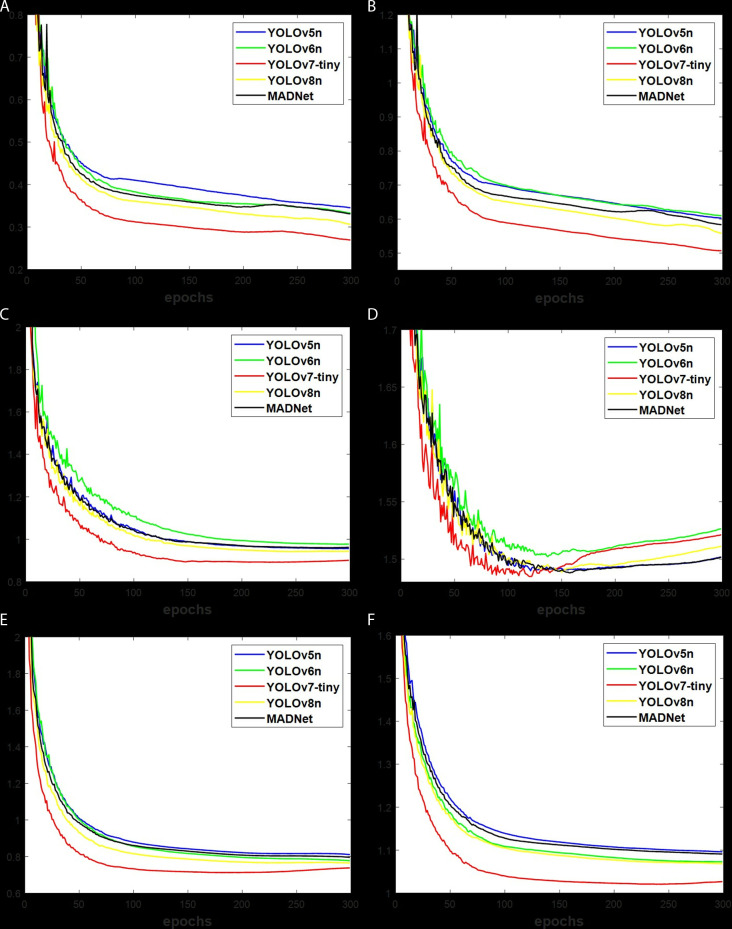
The anchor-free validation loss for (a) Brackish-Clsloss, (b) Brackish-Bbloss, (c) UOv2-Clsloss, (d) UOv2-Bbloss, (e) RUOD-Clsloss, and (f) RUOD-Bbloss.

The validation loss in Fig 7 and Fig 8 indicates the level of errors being produced during algorithm training. This loss reduces along with the increase of the epochs during learning process. With similarity in loss decreasing trend, the Clsloss and Bbloss in Fig 7(a-7b) and Fig 8(a-8b) for the brackish dataset are lower than Fig 7(c-7d) and Fig 8c-8d) for the RUOD dataset, and Fig 7(e-7f) and Fig 8e-8f) for the UOv2 dataset due to the number of bounding boxes. According to Fig 7 and Fig 8, YOLOv7-tiny shows a deeper neural network with the smallest loss, followed by MADNet, YOLOv8n, YOLOv5n and YOLOv6n. The displayed figures indicate that the validation losses of anchor-based methods in Fig 7 are lower than those of anchor-free methods in Fig 8. This is as a result of the longer training time associated with anchor-based methods, as mentioned in Table 2.

### Accuracy

The obtained losses of algorithms stated in Fig 7 and Fig 8, particularly with the Bbloss, which measures the actual position of targets in an image, resulted in the depicted findings in Table 4 for anchor-based algorithms and Table 6 for anchor-free algorithms under a valid-set. But the test-set, the unseen data with results presented in Table 5 for anchor-based algorithms and Table 7 for anchor-free algorithms, provides an accurate performance compared to the valid-set. Having to use mAP set at 50% as a comparison tool is more accurate than P% and R% because it provides the overall values over multi-class according to Eq. (7). With reference to anchor-based methods in Table 4, the mAP of MADNet is 0.1%, 0.3%, 0.8% and -0.1% under brackish, 3.8%, 0.4%, 6.2% and -0.6% under UOv2, and 0.1%, 0.1%, 0.3% and -0.5% under RUOD datasets, respectively, more accurate than YOLOv8n, YOLOv5n, YOLOv6n and YOLOv7-tiny. For Table 5, the mAP of MADNet is 0.1%, 0.3%, 1.0% and -0.1% under brackish, -1.1%, 0.8%, 3.3% and -1.3% under UOv2, and 1.1%, 0.5%, 1.4% and 0.9% under RUOD datasets, respectively, more accurate than YOLOv8n, YOLOv5n, YOLOv6n and YOLOv7-tiny. Using the anchor-free methods of Table 6, the mAP of MADNet is 0.2%, 0.4%, 0.6% and -0.1% under brackish, 0.9%, 4.7%, 3.5% and 0.1% under UOv2, and 0.6%, 0.9%, 2.2% and -0.5% under RUOD datasets, respectively, more accurate than YOLOv8n, YOLOv5n, YOLOv6n and YOLOv7-tiny. In the case of Table 7, the mAP of MADNet is 0.9%, 0.6%, 1.0% and -0.2% under brackish, 4.2%, 4.4%, 7.6% and -13.1% under UOv2, and 1.1%, 0.7%, 1.7% and 0.4% under RUOD datasets, respectively, more accurate than YOLOv8n, YOLOv5n, YOLOv6n and YOLOv7-tiny. This demonstrated the superior mAP of MADNet compared to YOLOv8n, YOLOv5n and YOLOv6n, but not for YOLOv7-tiny. However, the YOLOv7-tiny is limited by the high computation costs, as stated in Table 2 and Table 3. The mAP analysis shows that anchor-free methods are more accurate than anchor-based ones. Additionally, the mAP of MADNet was noted to be more accurate than improved YOLOv5 [28–29], MAD-YOLO [30], improved B-YOLOX-S [31], YOLOv6-ESG [35], and YOLOv7-AC [34] using similar brackish and UOv2 datasets.

**Table 4:** The accuracy of anchor-based algorithms using valid-set.

Algorithms	Brackish			UOv2			RUOD		
(valid)	P%	R%	mAP%	P%	R%	mAP%	P%	R%	mAP%
YOLOv5n	98.7	97.2	98.5	74.5	65.5	68.8	85.9	78.2	84.4
YOLOv6n	98.5	96.6	98.0	84.7	57.2	63.0	85.1	77.9	84.2
YOLOv7-tiny	98.8	98.3	98.9	69.1	66.5	69.8	86.6	79.9	85.0
YOLOv8n	98.6	97.3	98.7	64.5	58.4	65.4	85.2	78.5	84.4
MADNet	99.1	97.2	98.8	70.7	65.3	69.2	85.6	78.6	84.5

**Table 5:** The accuracy of anchor-based algorithms using test-set.

Algorithms	Brackish			UOv2			RUOD		
(test)	P%	R%	mAP%	P%	R%	mAP%	P%	R%	mAP%
YOLOv5n	97.9	96.7	98.3	64.7	59.1	65.1	81.2	78.0	83.5
YOLOv6n	97.9	95.0	97.6	83.9	58.1	62.6	83.7	77.5	82.6
YOLOv7-tiny	98.9	97.8	98.7	62.6	71.5	67.2	80.2	77.5	83.1
YOLOv8n	98.7	96.4	98.5	60.7	71.2	67.0	82.4	77.6	82.9
MADNet	97.7	96.7	98.6	84.7	58.4	65.9	79.2	79.2	84.0

**Table 6:** The accuracy of anchor-free algorithms using valid-set.

Algorithms	Brackish			UOv2			RUOD		
(valid)	P%	R%	mAP%	P%	R%	mAP%	P%	R%	mAP%
YOLOv5n	98.2	97.0	98.5	72.5	64.2	67.9	86.0	77.2	84.7
YOLOv6n	96.9	97.2	98.3	62.5	71.0	69.1	85.3	76.1	83.4
YOLOv7-tiny	98.8	98.3	99.0	75.4	65.9	72.5	86.2	79.9	86.1
YOLOv8n	98.7	96.8	98.7	73.6	65.7	71.7	84.9	78.4	85.0
MADNet	98.9	97.5	98.9	78.1	63.7	72.6	85.8	79.7	85.6

**Table 7:** The accuracy of anchor-free algorithms using test-set.

Algorithms	Brackish			UOv2			RUOD		
(test)	P%	R%	mAP%	P%	R%	mAP%	P%	R%	mAP%
YOLOv5n	98.2	95.8	98.2	83.0	58.4	64.4	83.0	76.1	84.0
YOLOv6n	97.8	95.7	97.8	82.2	55.7	61.2	79.9	75.9	83.0
YOLOv7-tiny	98.9	97.6	99.0	58.7	69.6	65.2	81.9	80.3	84.3
YOLOv8n	98.6	96.1	97.9	63.9	67.8	64.6	86.2	73.8	83.6
MADNet	98.7	97.3	98.8	75.1	66.4	68.8	84.2	76.3	84.7

### Speed

The speed detection of anchor-based and anchor-free algorithms, respectively, evaluated on a valid set and test set using Eq. (10), is shown in Table 8 and 9. As shown in Table 8, the speed of MADNet is faster than YOLOv6n, YOLOv8n, YOLOv5n, and YOLOv7-tiny using the datasets of brackish, UOv2, and RUOD. This is to support the idea that the detection speed of algorithms depends on both computation cost and layers [8], with reference to Table 2. Meanwhile, the speed evaluated on brackish is faster than RUOD, followed by UOv2 datasets. According to Table 9 of the anchor-free method, the speed of MADNet is higher than YOLOv8n, YOLOv5n, and YOLOv7-tiny using all the underwater datasets, but lower than YOLOv6n. The faster speed of YOLOv6n compared to MADNet is attributed to it having the fewest layers, as shown in Table 3. The results obtained from comparing the speed of the anchor-based and anchor-free algorithms are not consistent, as shown in Table 8 and Table 9, indicating the need for further investigation. Moreover, the inference time of anchor-based methods is faster than anchor-free methods. Summarily, the anchor-based algorithms are faster than anchor-free algorithms using the brackish and UOv2 datasets; however, anchor-free algorithms are faster than anchor-based algorithms using the RUOD dataset. Additionally, MADNet is faster for real-time detection compared to other YOLO-variant algorithms such as improved YOLOv5 [28], MAD-YOLO [30], YOLOv6-ESG [35], YOLOv7-AC [34], and so on.

**Table 8:** The speed of anchor-based algorithms.

Algorithms (speed)	Brackish				UOv2				RUOD			
	T_pre_	T_infer_	T_post_	fps	T_pre_	T_infer_	T_post_	fps	T_pre_	T_infer_	T_post_	fps
YOLOv5n	0.2	1.2	0.8	455	0.3	2.3	0.8	294	0.2	2.1	0.8	323
YOLOv6n	0.2	1.2	0.7	476	0.3	2.0	0.8	323	0.2	2.0	0.8	333
YOLOv7-tiny	0.2	1.9	0.8	345	0.3	3.4	0.8	222	0.2	3.3	0.9	227
YOLOv8n	0.2	1.2	0.8	455	0.3	2.2	0.9	294	0.2	1.8	0.9	345
MADNet	0.2	1.1	0.7	500	0.3	1.8	0.8	345	0.2	1.8	0.7	370

**Table 9:** The speed of anchor-free algorithms.

Algorithms (speed)	Brackish				UOv2				RUOD			
	T_pre_	T_infer_	T_post_	fps	T_pre_	T_infer_	T_post_	fps	T_pre_	T_infer_	T_post_	fps
YOLOv5n	0.2	1.5	1.0	370	0.3	2.3	0.9	286	0.3	1.3	1.2	357
YOLOv6n	0.2	1.2	0.9	435	0.3	2.0	0.8	323	0.3	0.8	1.0	476
YOLOv7-tiny	0.2	2.7	0.9	263	0.3	4.6	0.6	182	0.3	1.6	1.3	313
YOLOv8n	0.2	1.5	0.9	385	0.3	2.3	0.8	294	0.3	1.1	1.2	385
MADNet	0.2	1.4	0.9	400	0.3	2.2	0.7	313	0.3	0.9	1.0	455

### Robustness

**Fig 9:**
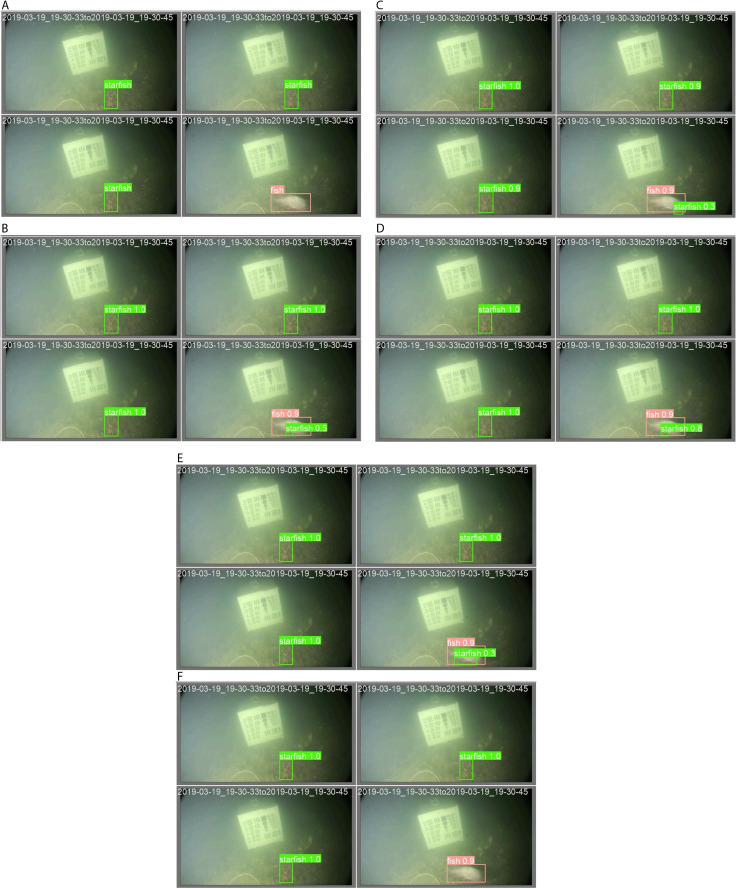
The displayed image’s (a) actual labels were taken from a valid set and detected using an anchor-based algorithm of (b) YOLOv5n, (c) YOLOv6n, (d) YOLOv7-tiny, (e) YOLOv8n, and (f) MADNet.

**Fig 10:**
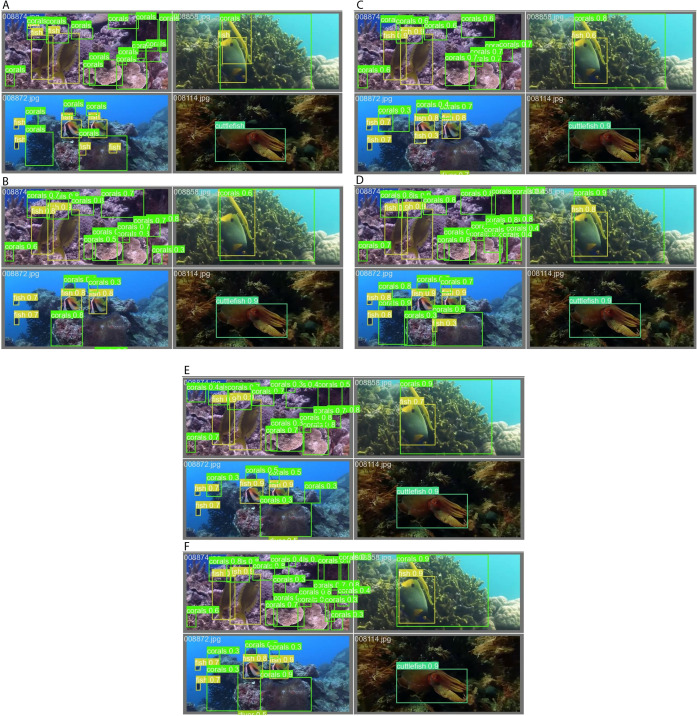
The displayed image’s (a) actual labels were taken from a valid set and detected using an anchor-free algorithm of (b) YOLOv5n, (c) YOLOv6n, (d) YOLOv7-tiny, (e) YOLOv8n, and (f) MADNet.

A number of marine animals were detected in images to assess robustness, as shown in Fig 9 (anchor-based methods) and Fig 10 (anchor-free methods). Nevertheless, the detected marine animals were associated with different confidence scores, missed detection, and incorrect detection, as shown in the figures. For example, the ground-truth annotations in Fig 9(a) are correct with Fig 9(f) of MADNet, unlike Fig 9(b) of YOLOv5n, Fig 9(c) of YOLOv6n, Fig 9(d) of YOLOv7-tiny, and Fig 9(e) of YOLOv8n, which are associated with incorrect detections. Because it is very difficult to quantify the number of counts for detected marine animals, just like in Fig 10, the MAE of Eq. (11) was introduced and analyzed based on the combination of the valid-set and test-set.

The derived MAE based on anchor-based methods estimates 0.071 of MADNet, 0.073 of YOLOv7-tiny, 0.079 of YOLOv8n, 0.084 of YOLOv5n, and 0.011 of YOLOv6n using the brackish dataset; 0.067 of MADNet, 0.062 of YOLOv7-tiny, 0.071 of YOLOv8n, 0.070 of YOLOv5n, and 0.092 of YOLOv6n using the UOv2 dataset; and 0.041 of MADNet, 0.028 of YOLOv7-tiny, 0.045 of YOLOv8n, 0.045 of YOLOv5n, and 0.058 of YOLOv6n using the RUOD dataset. This demonstrated a lower error of count performance for MADNet compared to other YOLO algorithms using brackish datasets, but not with YOLOv7-tiny using both UOv2 and RUOD datasets. Similarly, the MAE obtained based on the anchor-free method indicates 0.006 of MADNet, 0.003 of YOLOv7-tiny, 0.007 of YOLOv8n, 0.012 of YOLOv5n, and 0.013 of YOLOv6n using the brackish dataset; 0.061 of MADNet, 0.040 of YOLOv7-tiny, 0.062 of YOLOv8n, 0.063 of YOLOv5n, and 0.087 of YOLOv6n using the UOv2 dataset; and 0.022 of MADNet, 0.014 of YOLOv7-tiny, 0.023 of YOLOv8n, 0.027 of YOLOv5n, and 0.034 of YOLOv6n using the RUOD dataset. The counting performance error of MADNet was observed to be the lowest value against other YOLO algorithms using the UOv2 dataset, but it happened to be second place after YOLOv7-tiny using the brackish and RUOD datasets. Meanwhile, the MAE values of anchor-free algorithms are lower than those of anchor-based algorithms, and the level of MAE is measured as YOLOv7-tiny is smaller than MADNet followed by YOLOv8n, YOLOv5n and YOLOv6n.

### Comparisons

Table 10 provides the choice for the best-selected algorithm between anchor-based and anchor-free in terms of computation cost and trained time from Table 2 and 3, training loss from Fig 7 and 8, accuracy from Table 4–7, speed from Table 8 and 9, and MAE from Eq. (11). With loss (shaded) excluded from Table 10 and just 1 and 5 used to calculate the performance score based on the positive side, the total performance score of anchor-free algorithms is 17 (56.7%), whereas that of anchor-based algorithms is 13 (43.3%). This affirmed the superiority and robustness of anchor-free methods over anchor-based methods for marine animal detection. Furthermore, the performance taken from a score range of 1–5 and analyzed shows that the total performance score of MADNet is 25 at 27.8%, YOLOv8n is 18 at 20%, YOLOv6n is 17 at 18.9%, YOLOv5n is 16 at 17.8%, and YOLOv7-tiny is 14 at 15.6%, according to Table 11. For these reasons, MADNet is robust against complex marine scenarios, has fewer parameters and computation costs, is accurate and fast, and is deployable, lightweight, and applicable for generalization.

**Table 10:** The performance comparison between anchor-based and anchor-free algorithms.

Methods	Computation cost	Trained time	Loss	Accuracy	Speed	MAE	Total
Anchor-based	Lowest: 5	Highest: 1	Lowest: 5	Lowest: 1	Highest: 5	Highest: 1	13
Anchor-free	Highest: 1	Lowest: 5	Highest: 1	Highest: 5	Lowest: 1	Lowest: 5	17

**Table 11:** The overall performance score comparison between algorithms.

Algorithms	Computation cost	Trained time	Loss	Accuracy	Speed	MAE	Total
YOLOv5n	Lower: 4	Higher: 2	Higher: 2	Lower: 2	Lower: 2	Higher: 4	16
YOLOv6n	Higher: 2	Lower: 4	Highest: 1	Lowest: 1	Higher: 4	Highest: 5	17
YOLOv7-tiny	Highest: 1	Highest: 1	Lowest: 5	Highest: 5	Lowest: 1	Lowest: 1	14
YOLOv8n	Medium: 3	Medium: 3	Medium: 3	Medium: 3	Medium: 3	Medium: 3	18
MADNet	Lowest: 5	Lowest: 5	Lower: 4	Higher: 4	Highest: 5	Lower: 2	25

## Conclusions and future plans

In the quest to achieve fewer parameters, low computation costs, high accuracy, high speed, lightweight deployment on low-power devices, and robustness against complex underwater environments, the MADNet algorithm was proposed based on the YOLO architecture. The architecture of YOLOv5 for anchor-based and YOLOv8 for anchor-free was selected for improvement to address the difficulties encountered in real-life underwater scenarios. Experiments were conducted on publicly available underwater image datasets, namely brackish, UOv2, and RUOD, to demonstrate the effectiveness of the algorithm and compare it to YOLOv7-tiny, YOLOv8n, YOLOv5n, and YOLOv6n. First, the cumulative performance score shows that the application of 56.7% for anchor-free methods exceeds 43.3% for anchor-based methods, making anchor-free methods the best choice. Secondly, the total performance scores taken from anchor-based and anchor-free methods in terms of weight-size, computation cost, trained time, loss, accuracy, speed, and mean absolute error (MAE) indicated the outstanding records of MADNet at 27.8% compared to YOLOv8n at 20%, YOLOv6n at 18.9%, YOLOv5n at 17.8%, and YOLOv7-tiny at 15.6%. Finally, MADNet is lightweight for deployment, applicable for generalization, robust, accurate and fast for marine animal detection to monitor underwater activities. Future investigations would require incorporating lightweight attention modules into the backbone to focus on salient features, improving detection in complex scenes while maintaining real-time performance and reducing the mean absolute error (MAE) of the MADNet when specifically evaluated on the UOv2 dataset.

## Supporting information

S1 File The relevant codes that support this study. (ZIP)
