# Supplementary figures and images for: MADNet: Marine Animal Detection Network using the YOLO platform
Source: PLoS One. 2025 May 8;20(5):e0322799. doi: 10.1371/journal.pone.0322799 (PMC12061432; doi:10.1371/journal.pone.0322799)

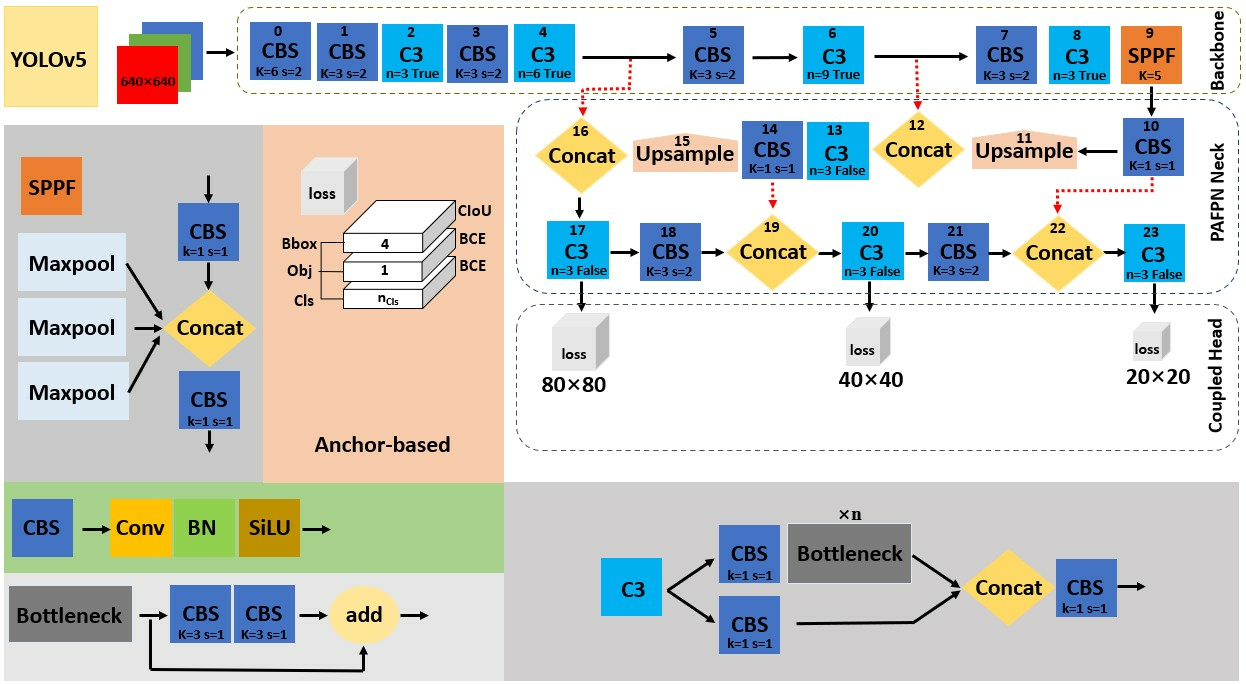

Supplement: S1 File — (ZIP) [file pone.0322799.s001.zip › Supporting information/PACE Corrected-Figures/Fig 1.tif]

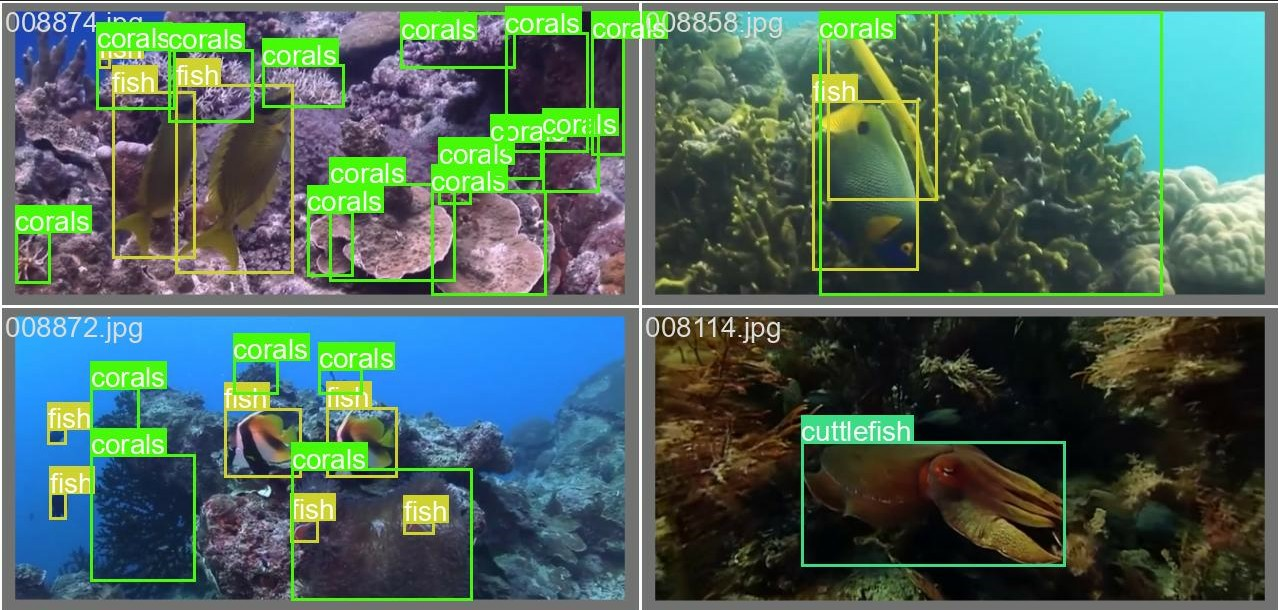

Supplement: S1 File — (ZIP) [file pone.0322799.s001.zip › Supporting information/PACE Corrected-Figures/Fig 10(a).tif]

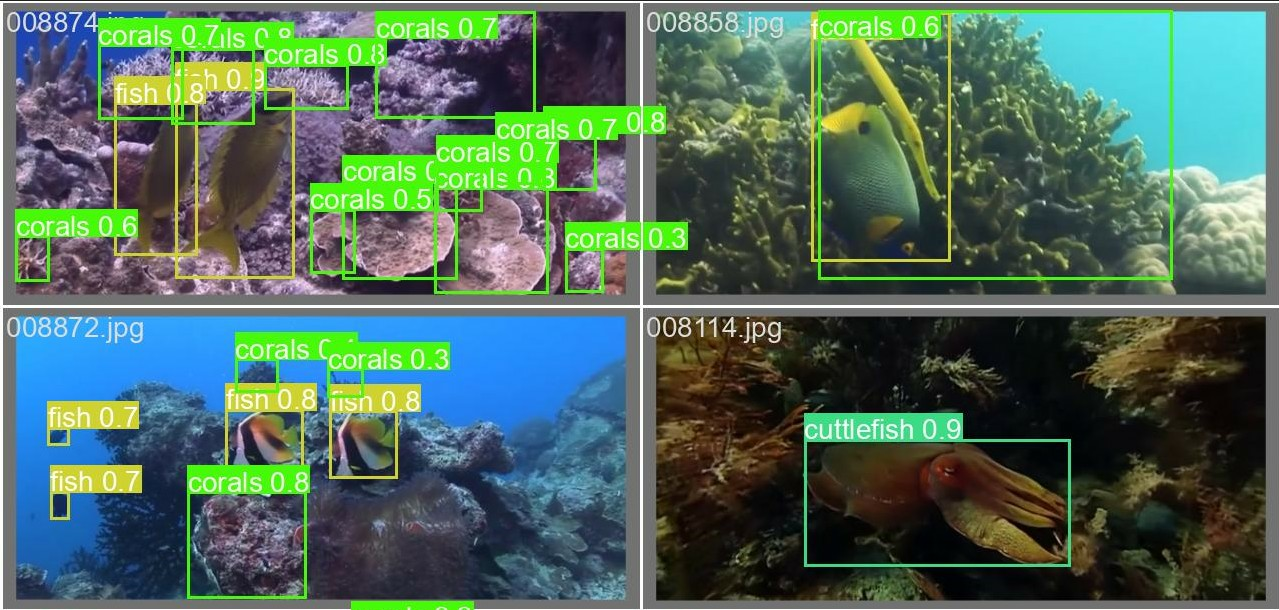

Supplement: S1 File — (ZIP) [file pone.0322799.s001.zip › Supporting information/PACE Corrected-Figures/Fig 10(b).tif]

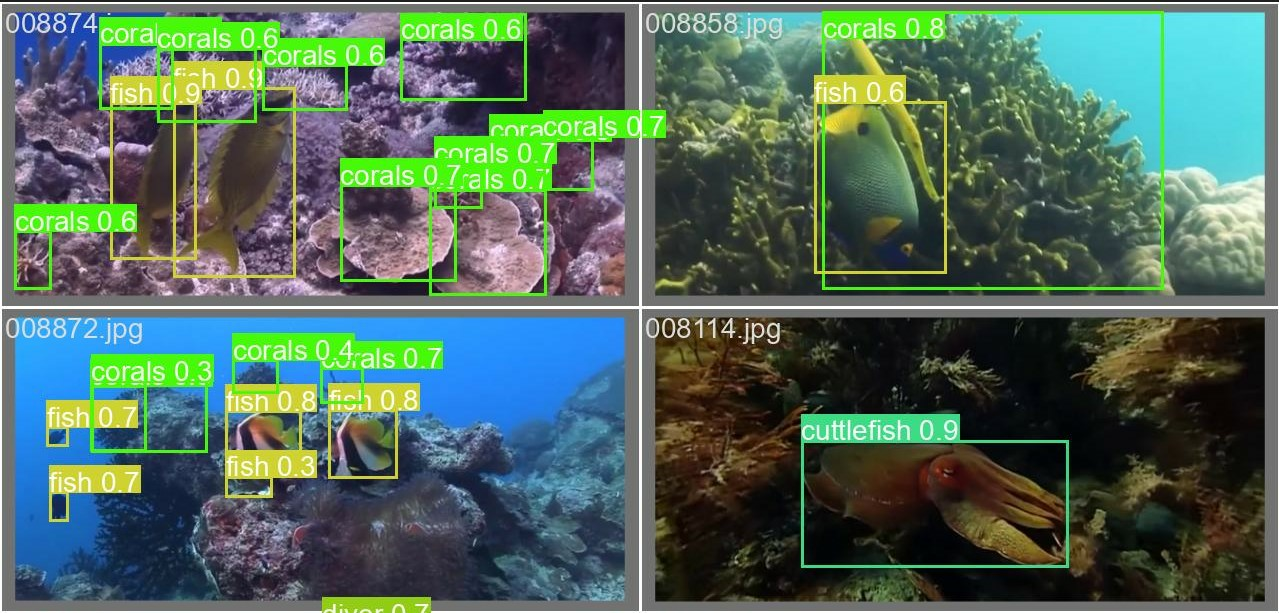

Supplement: S1 File — (ZIP) [file pone.0322799.s001.zip › Supporting information/PACE Corrected-Figures/Fig 10(c).tif]

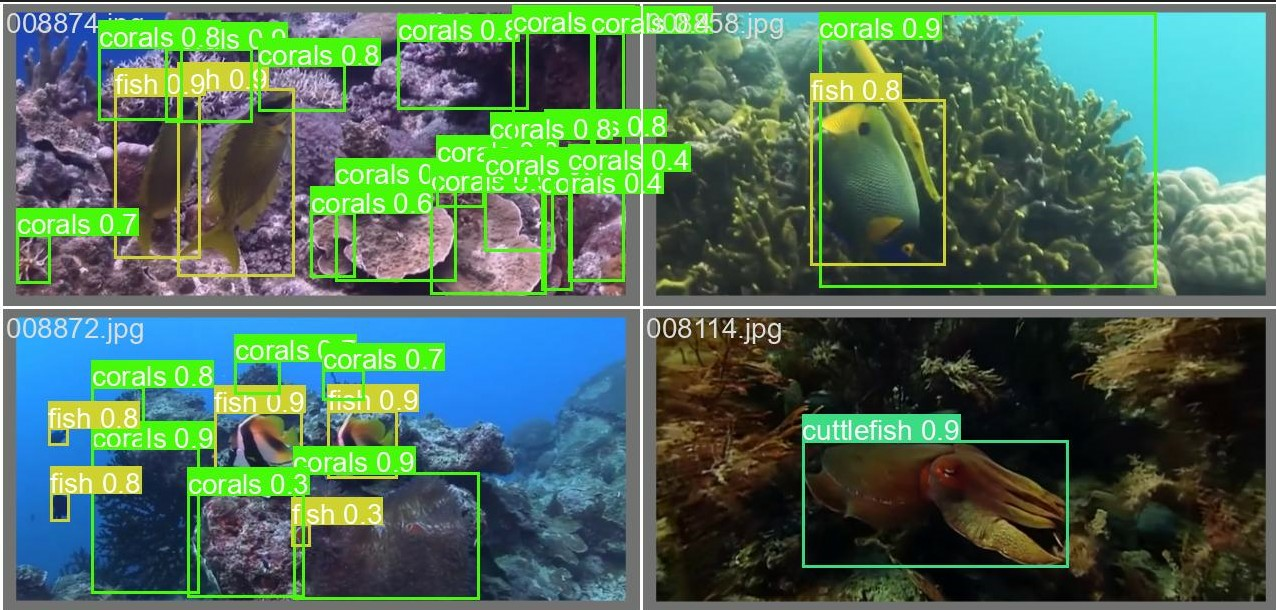

Supplement: S1 File — (ZIP) [file pone.0322799.s001.zip › Supporting information/PACE Corrected-Figures/Fig 10(d).tif]

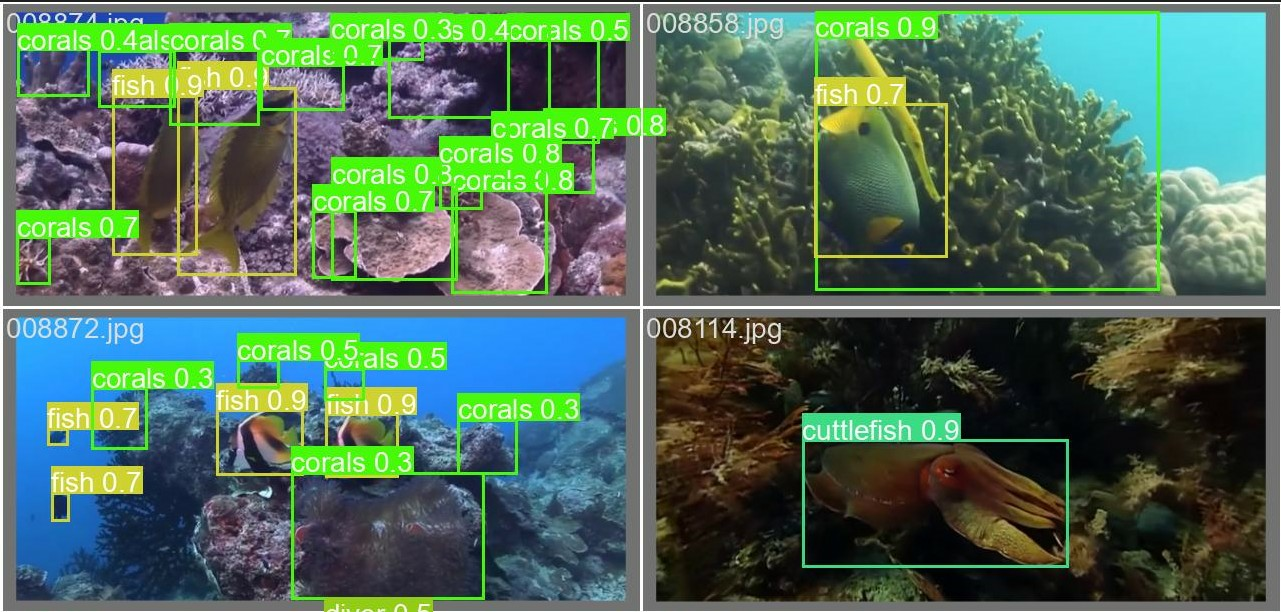

Supplement: S1 File — (ZIP) [file pone.0322799.s001.zip › Supporting information/PACE Corrected-Figures/Fig 10(e).tif]

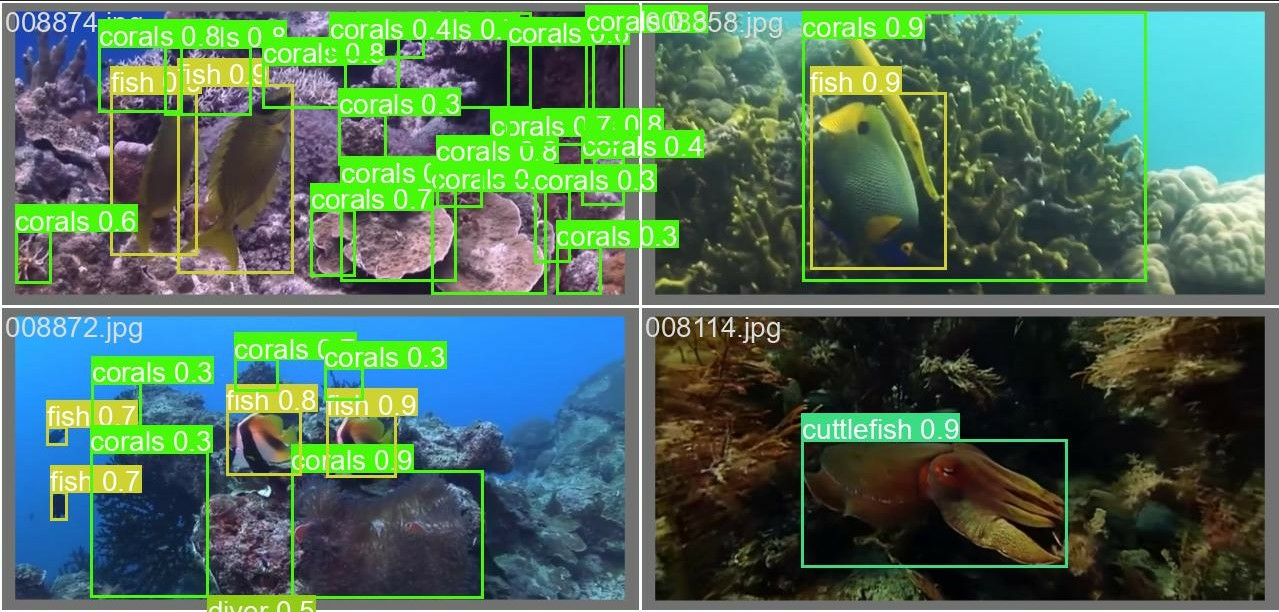

Supplement: S1 File — (ZIP) [file pone.0322799.s001.zip › Supporting information/PACE Corrected-Figures/Fig 10(f).tif]

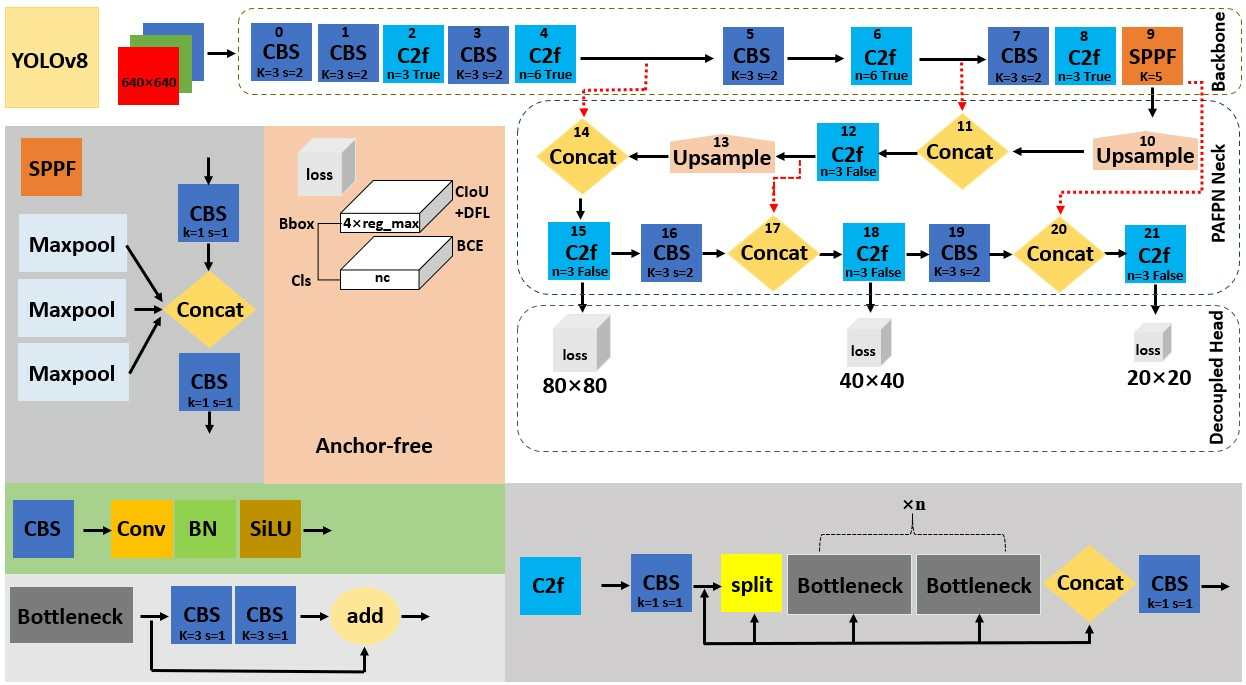

Supplement: S1 File — (ZIP) [file pone.0322799.s001.zip › Supporting information/PACE Corrected-Figures/Fig 2.tif]

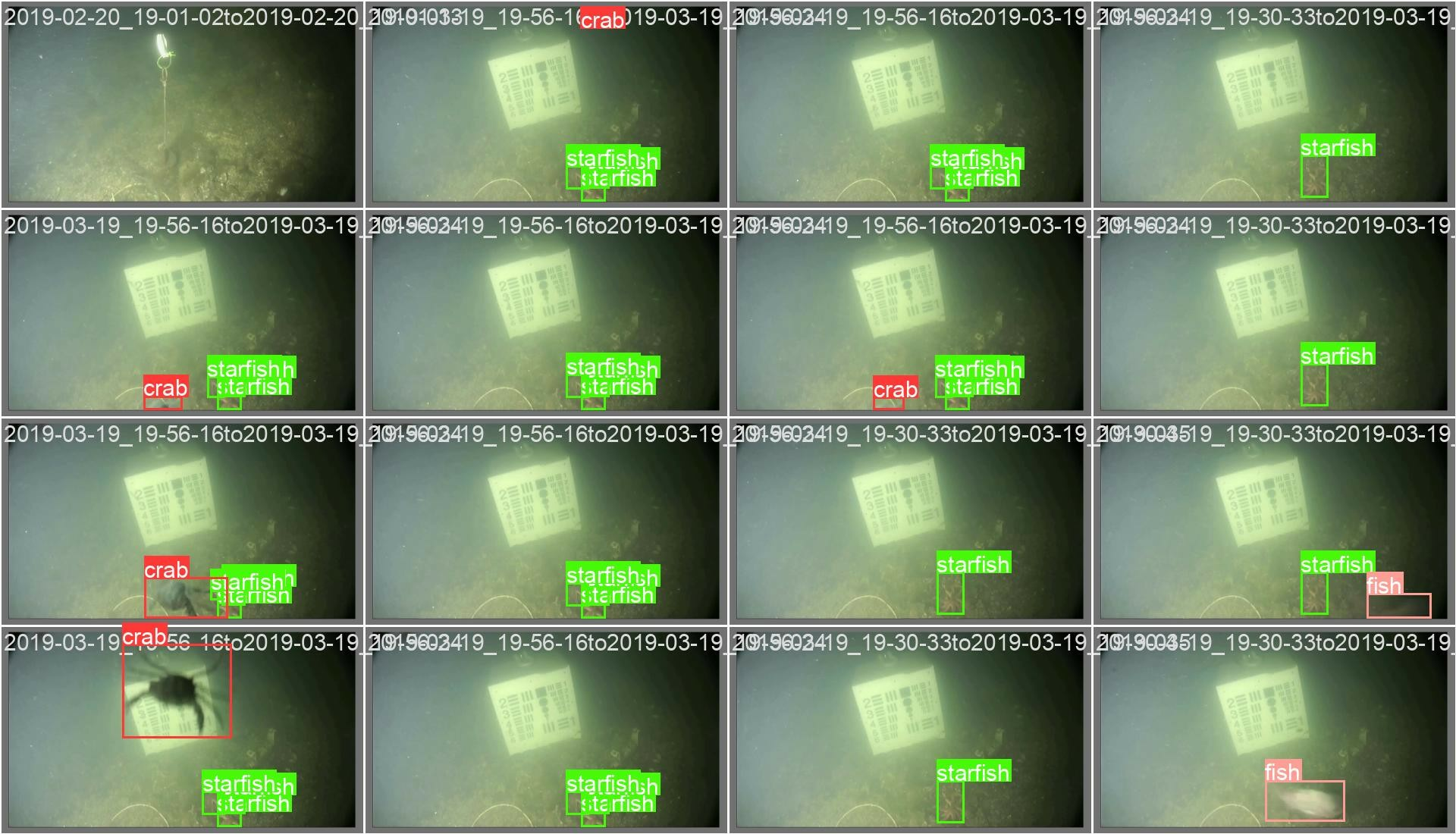

Supplement: S1 File — (ZIP) [file pone.0322799.s001.zip › Supporting information/PACE Corrected-Figures/Fig 3.tif]

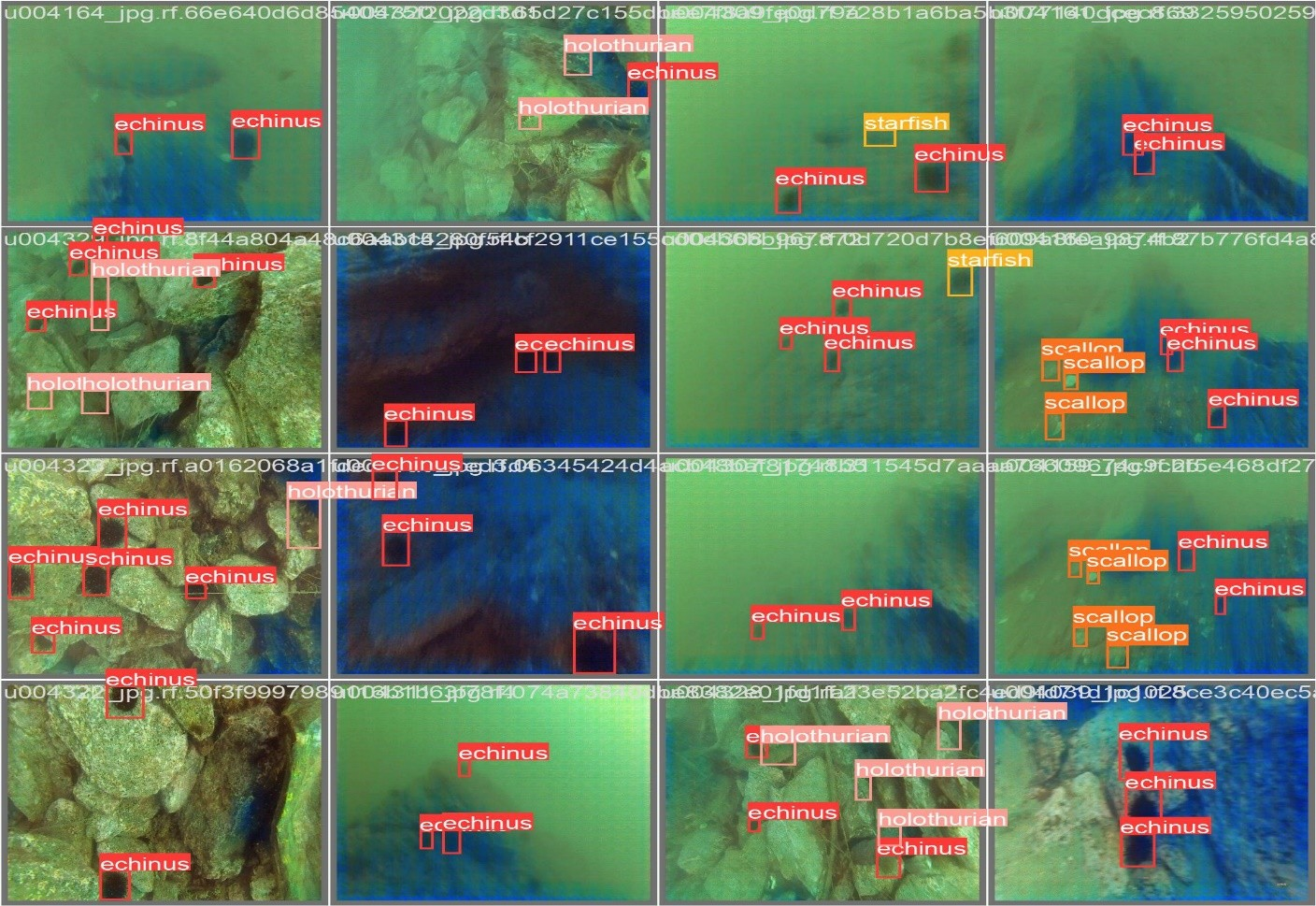

Supplement: S1 File — (ZIP) [file pone.0322799.s001.zip › Supporting information/PACE Corrected-Figures/Fig 4.tif]

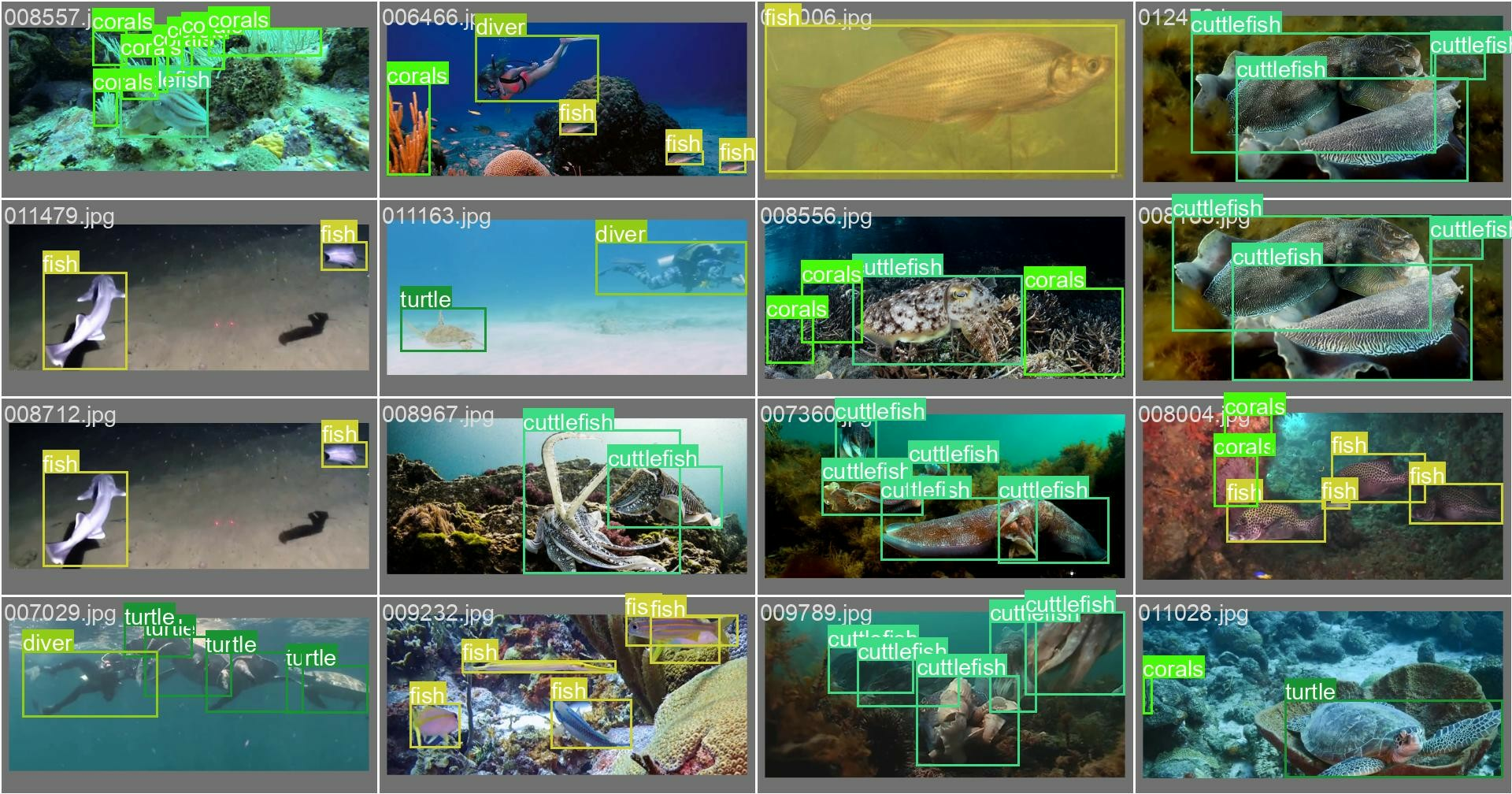

Supplement: S1 File — (ZIP) [file pone.0322799.s001.zip › Supporting information/PACE Corrected-Figures/Fig 5.tif]

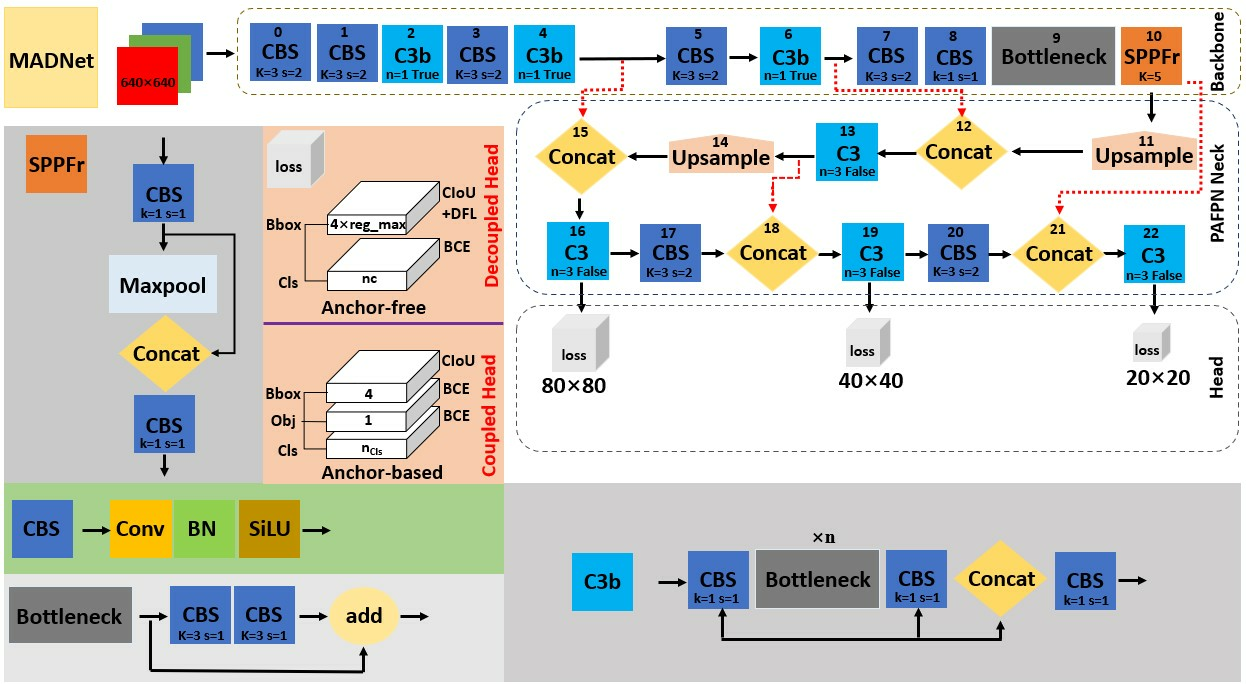

Supplement: S1 File — (ZIP) [file pone.0322799.s001.zip › Supporting information/PACE Corrected-Figures/Fig 6.tif]

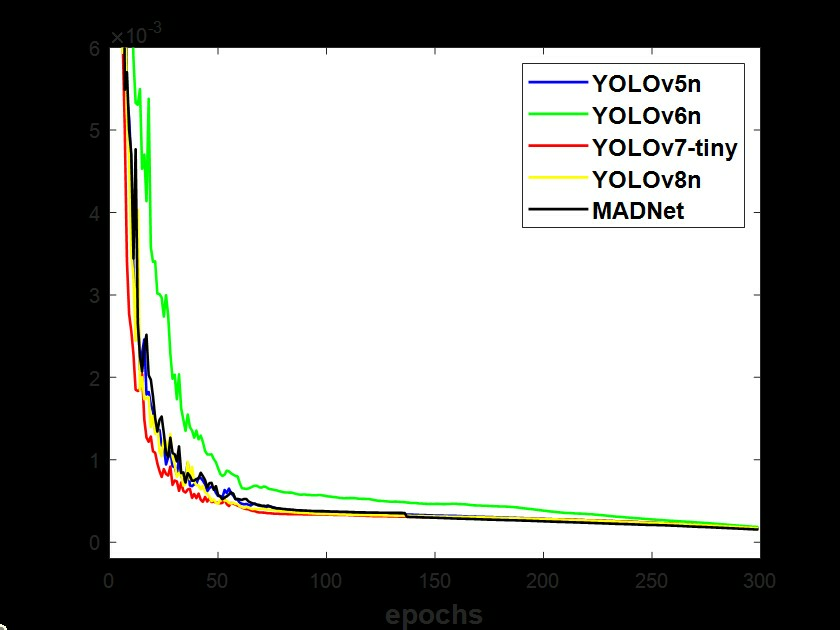

Supplement: S1 File — (ZIP) [file pone.0322799.s001.zip › Supporting information/PACE Corrected-Figures/Fig 7(a).tif]

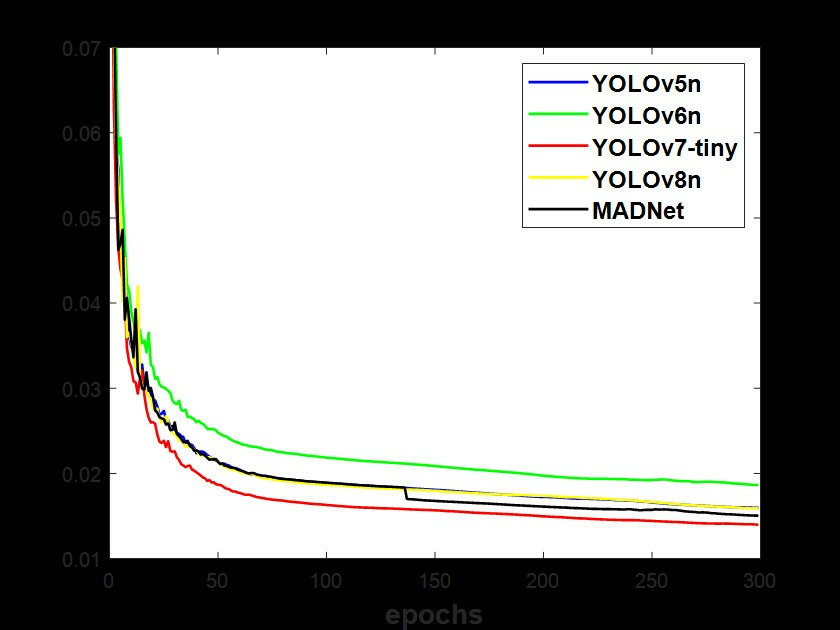

Supplement: S1 File — (ZIP) [file pone.0322799.s001.zip › Supporting information/PACE Corrected-Figures/Fig 7(b).tif]

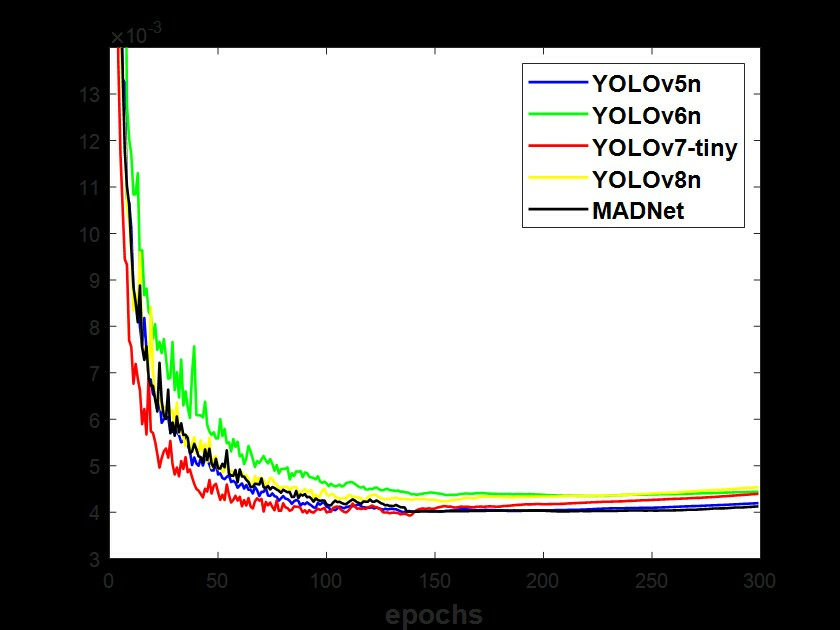

Supplement: S1 File — (ZIP) [file pone.0322799.s001.zip › Supporting information/PACE Corrected-Figures/Fig 7(c).tif]

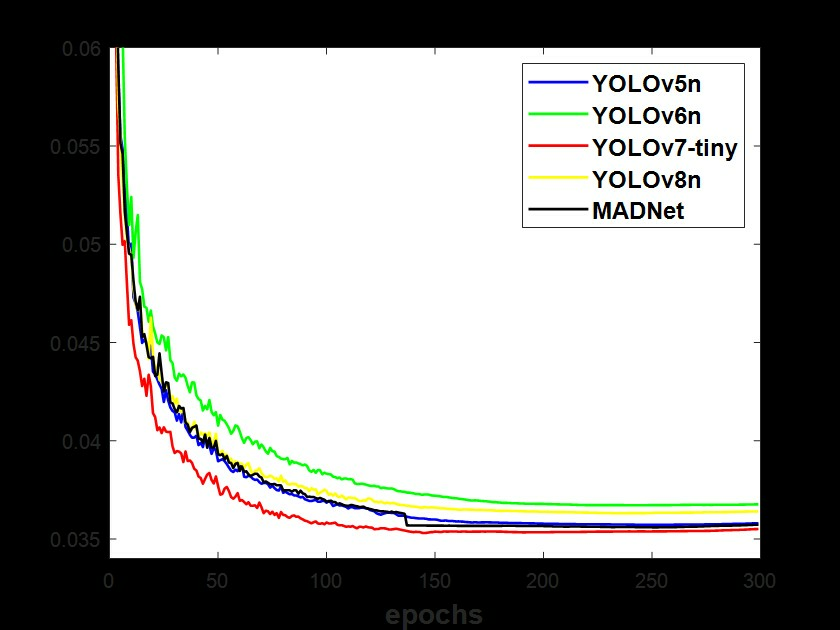

Supplement: S1 File — (ZIP) [file pone.0322799.s001.zip › Supporting information/PACE Corrected-Figures/Fig 7(d).tif]

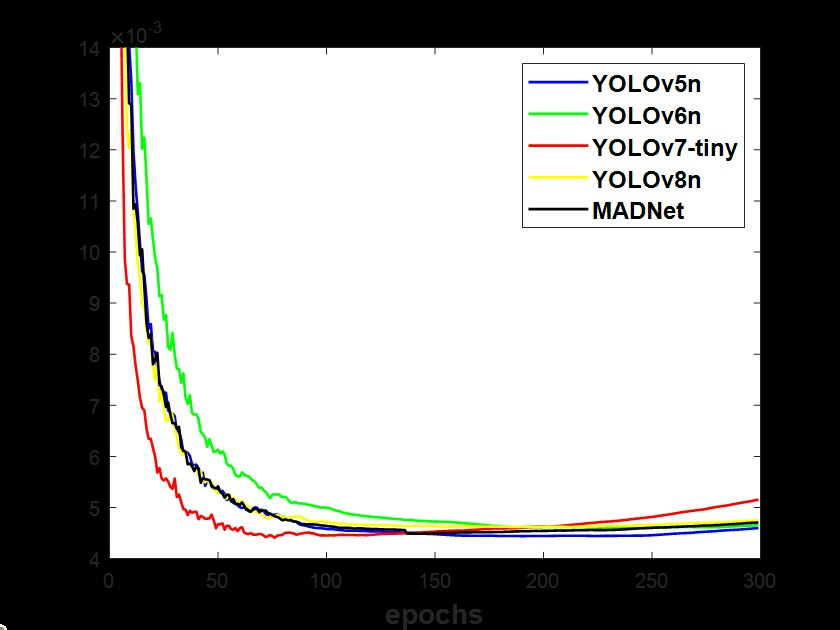

Supplement: S1 File — (ZIP) [file pone.0322799.s001.zip › Supporting information/PACE Corrected-Figures/Fig 7(e).tif]

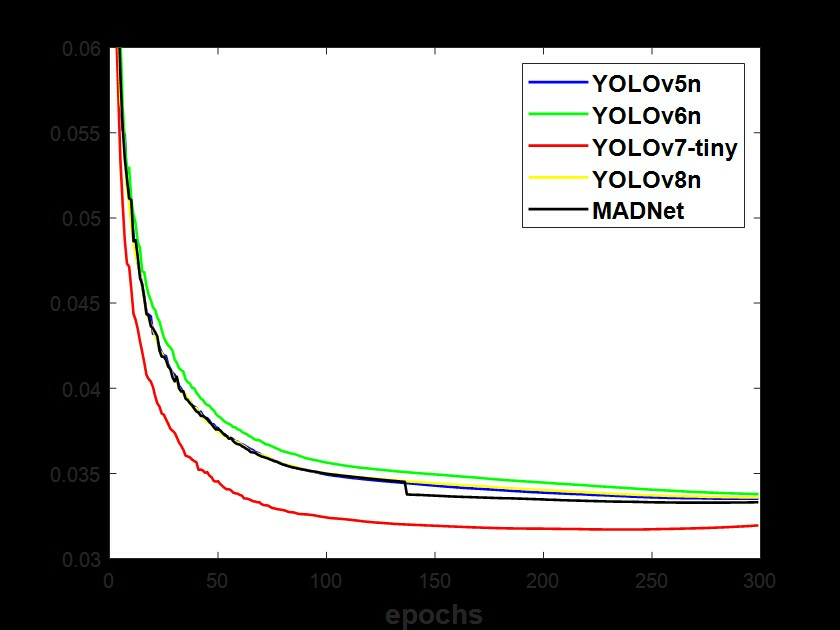

Supplement: S1 File — (ZIP) [file pone.0322799.s001.zip › Supporting information/PACE Corrected-Figures/Fig 7(f).tif]

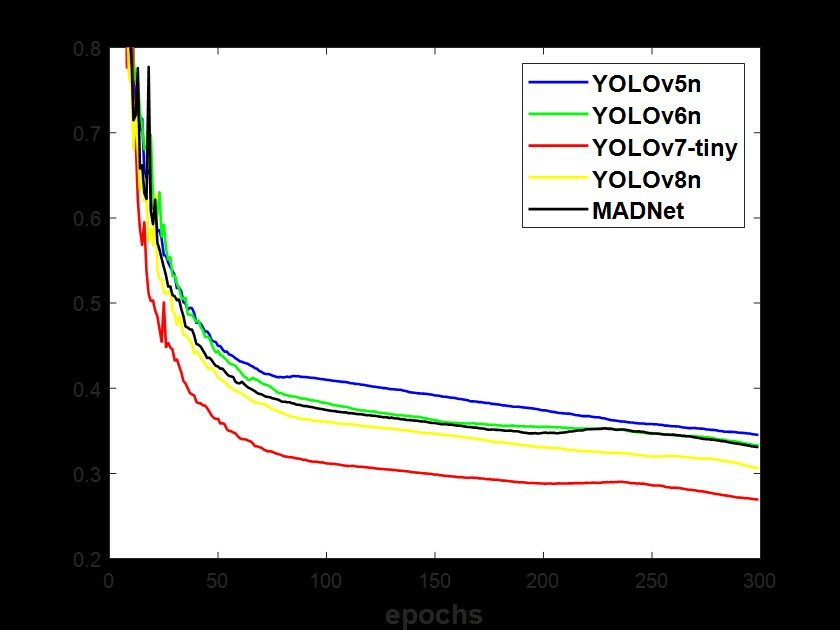

Supplement: S1 File — (ZIP) [file pone.0322799.s001.zip › Supporting information/PACE Corrected-Figures/Fig 8(a).tif]

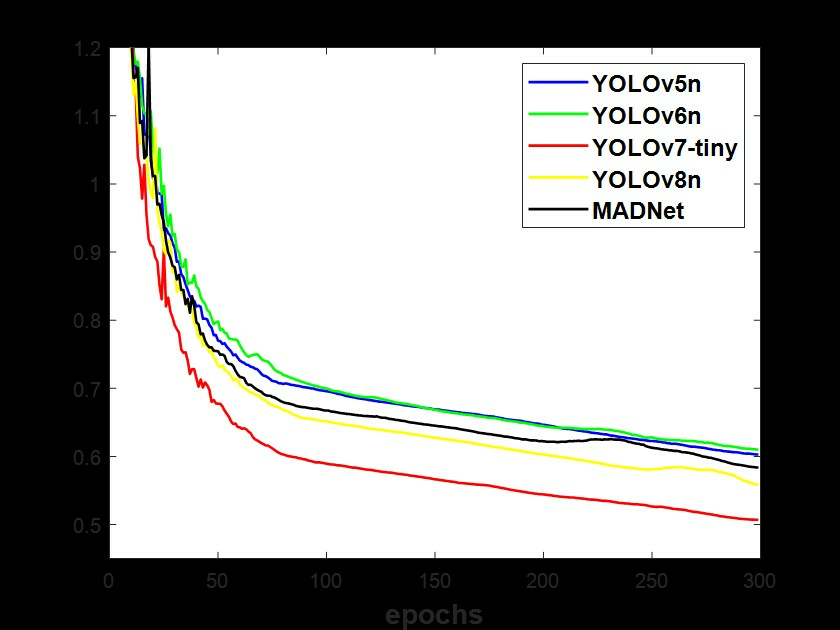

Supplement: S1 File — (ZIP) [file pone.0322799.s001.zip › Supporting information/PACE Corrected-Figures/Fig 8(b).tif]

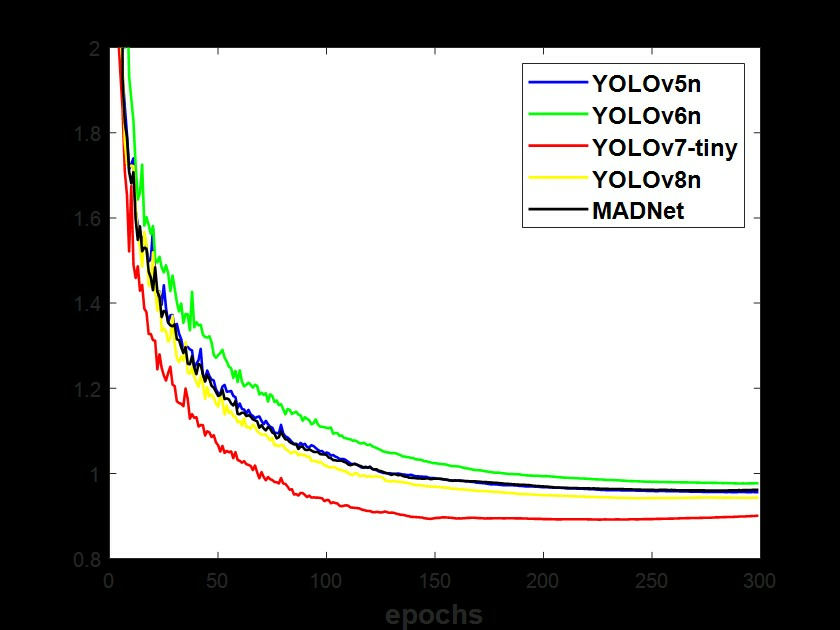

Supplement: S1 File — (ZIP) [file pone.0322799.s001.zip › Supporting information/PACE Corrected-Figures/Fig 8(c).tif]

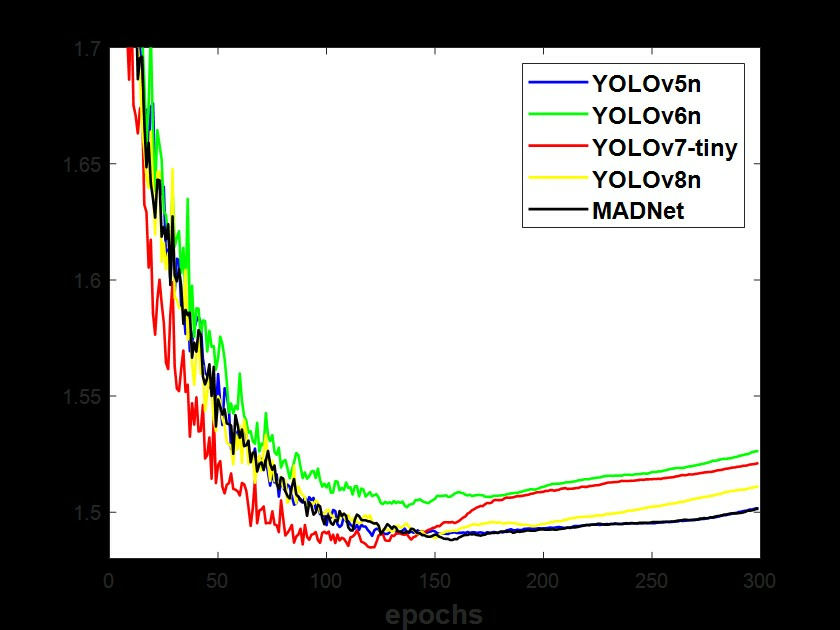

Supplement: S1 File — (ZIP) [file pone.0322799.s001.zip › Supporting information/PACE Corrected-Figures/Fig 8(d).tif]

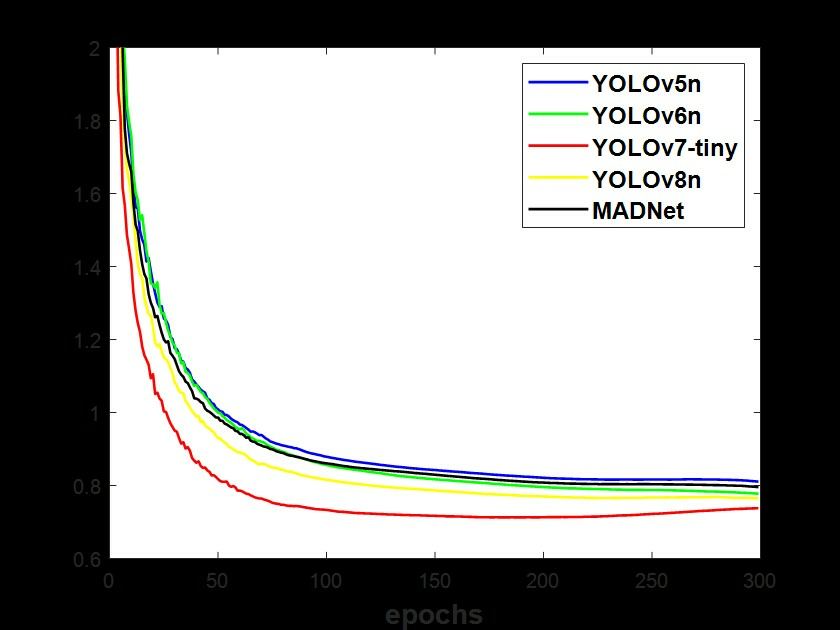

Supplement: S1 File — (ZIP) [file pone.0322799.s001.zip › Supporting information/PACE Corrected-Figures/Fig 8(e).tif]

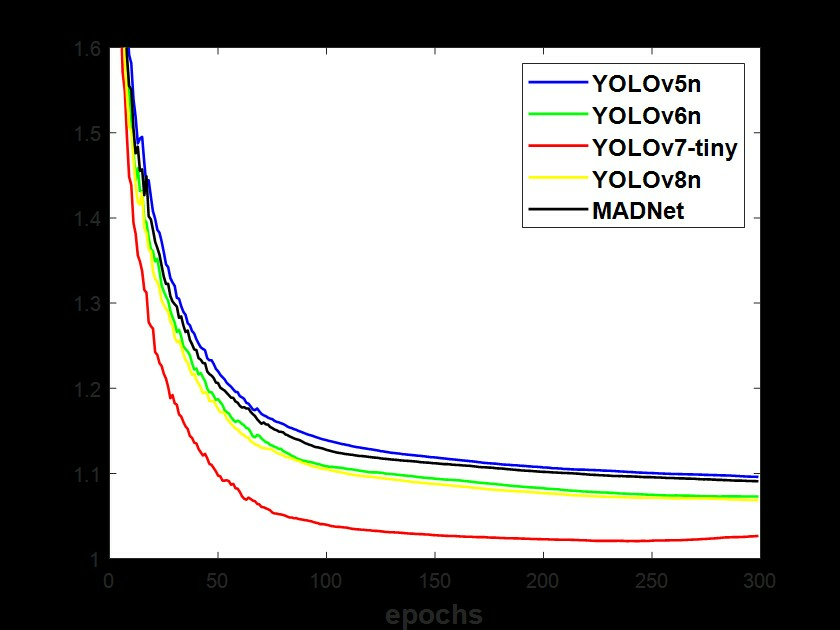

Supplement: S1 File — (ZIP) [file pone.0322799.s001.zip › Supporting information/PACE Corrected-Figures/Fig 8(f).tif]

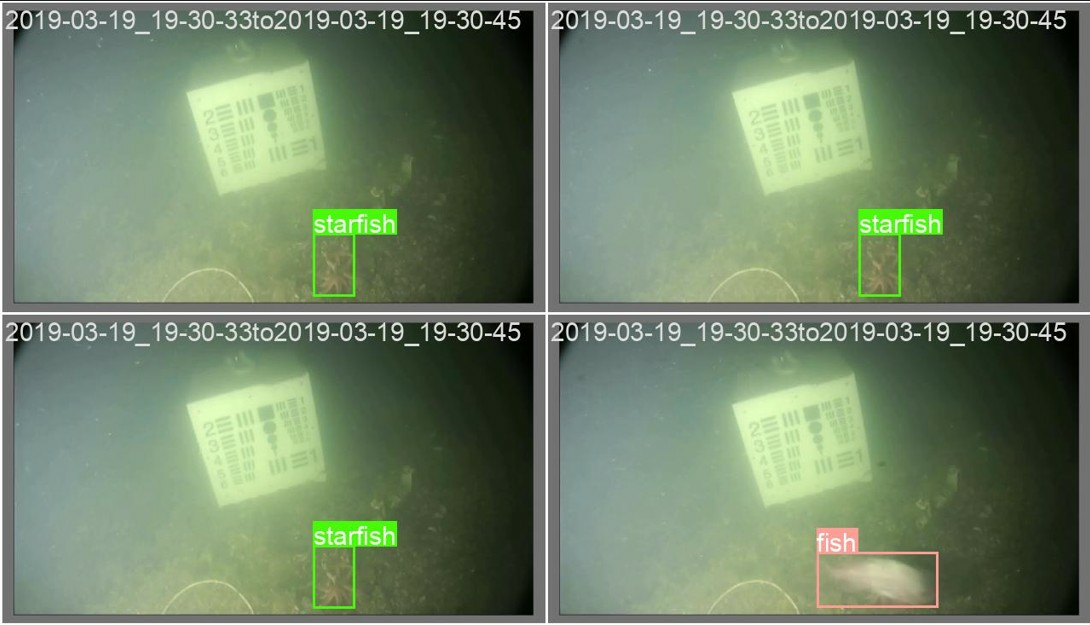

Supplement: S1 File — (ZIP) [file pone.0322799.s001.zip › Supporting information/PACE Corrected-Figures/Fig 9(a).tif]

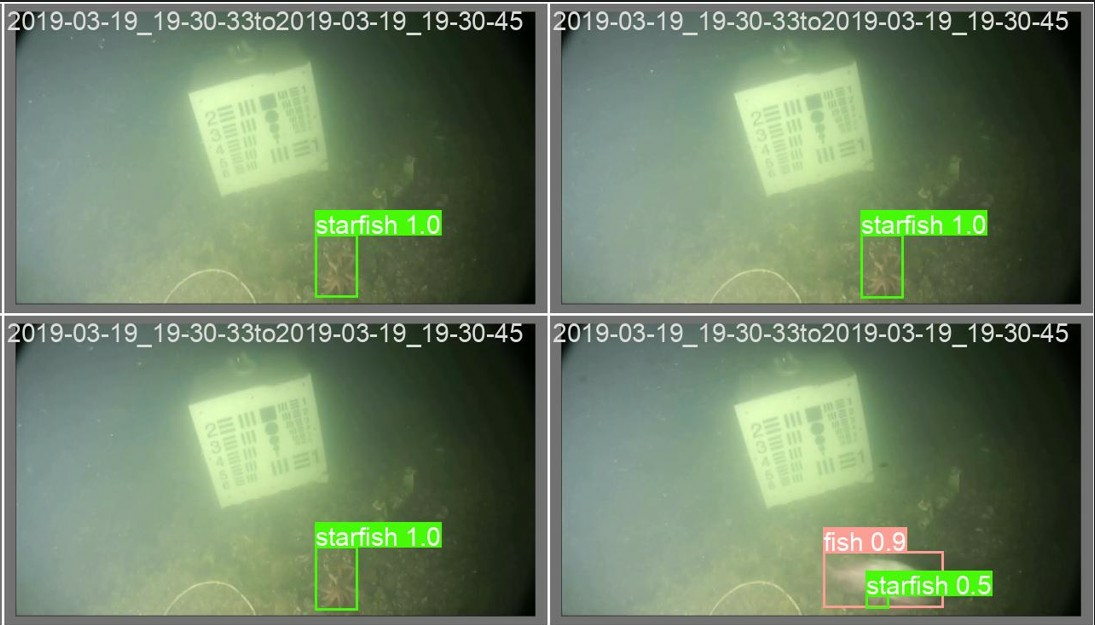

Supplement: S1 File — (ZIP) [file pone.0322799.s001.zip › Supporting information/PACE Corrected-Figures/Fig 9(b).tif]

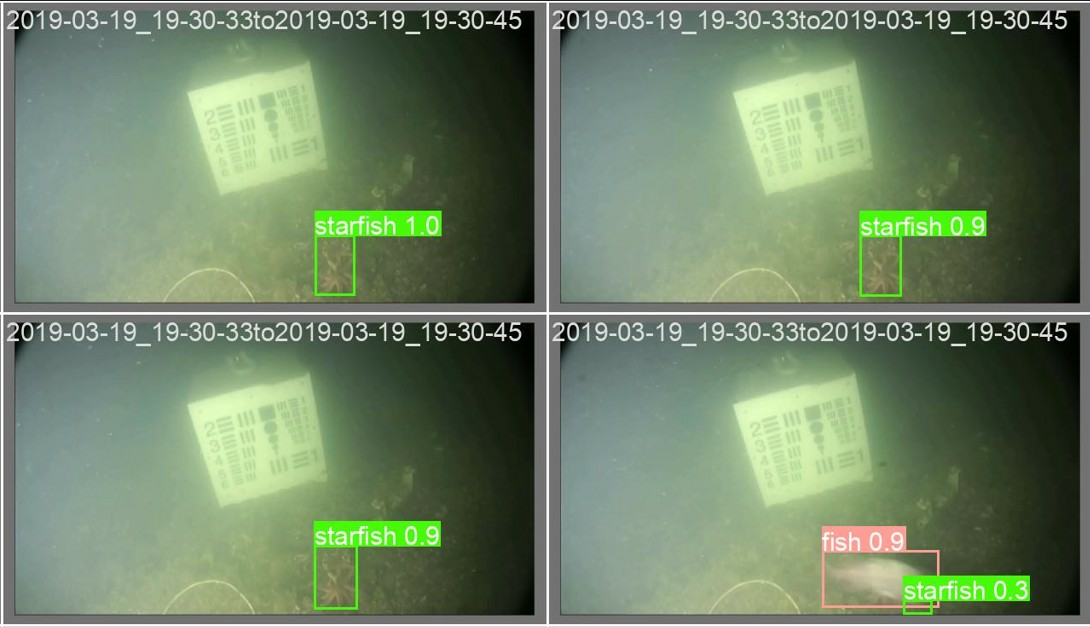

Supplement: S1 File — (ZIP) [file pone.0322799.s001.zip › Supporting information/PACE Corrected-Figures/Fig 9(c).tif]

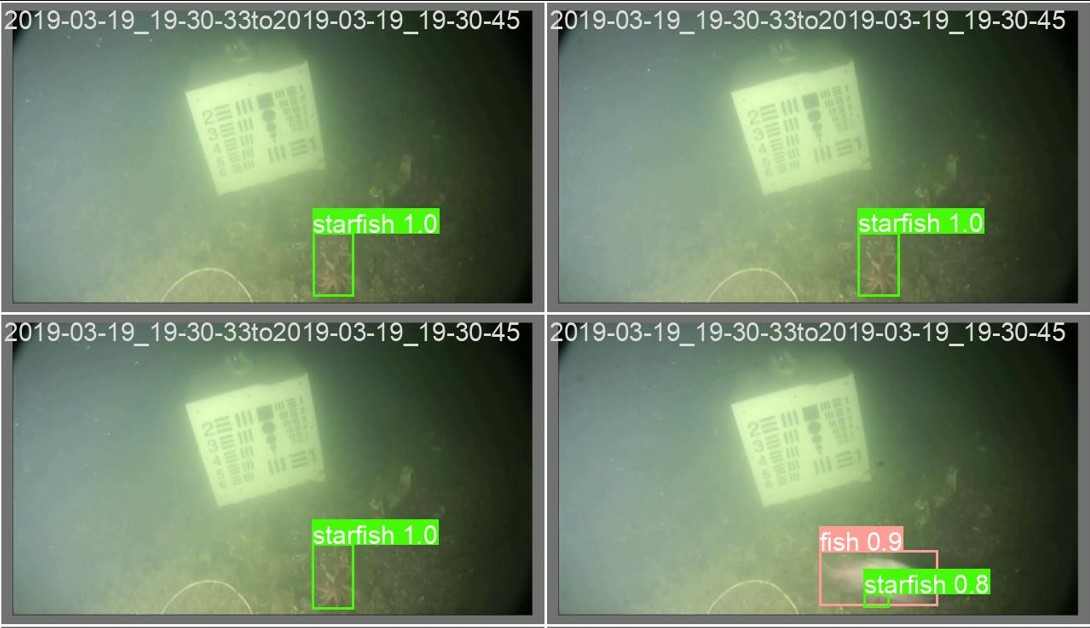

Supplement: S1 File — (ZIP) [file pone.0322799.s001.zip › Supporting information/PACE Corrected-Figures/Fig 9(d).tif]

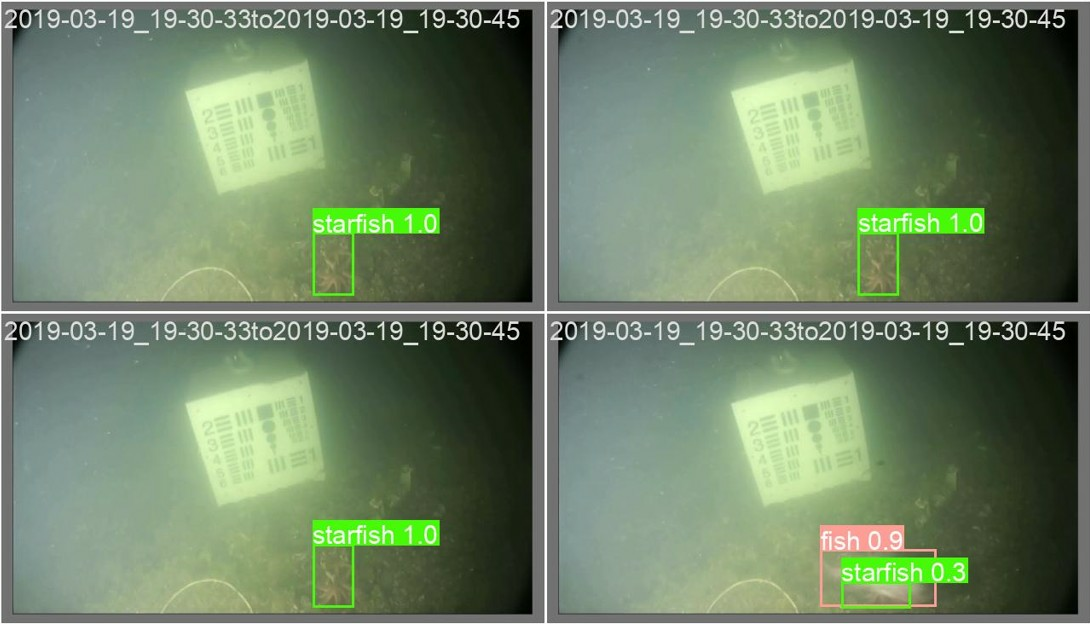

Supplement: S1 File — (ZIP) [file pone.0322799.s001.zip › Supporting information/PACE Corrected-Figures/Fig 9(e).tif]

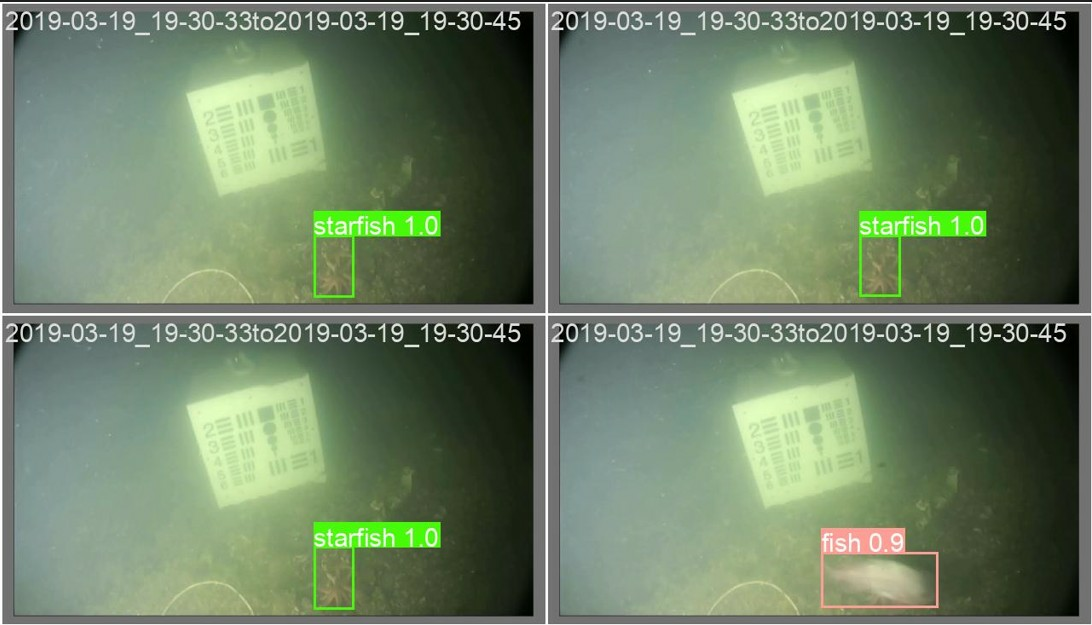

Supplement: S1 File — (ZIP) [file pone.0322799.s001.zip › Supporting information/PACE Corrected-Figures/Fig 9(f).tif]
